# Pseudo-superparamagnetic behaviour of barium hexaferrite particles†

**DOI:** 10.1039/d0ra01619e

**Published:** 2020-05-18

**Authors:** Szymon Dudziak, Zuzanna Ryżyńska, Zuzanna Bielan, Jacek Ryl, Tomasz Klimczuk, Anna Zielińska-Jurek

**Affiliations:** Department of Process Engineering and Chemical Technology, Gdansk University of Technology G. Narutowicza 11/12 80-233 Gdansk Poland dudziakszy@gmail.com; Faculty of Applied Physics and Mathematics and Advanced Materials Centre, Gdansk University of Technology Narutowicza 11/12 80-233 Gdansk Poland; Department of Electrochemistry, Corrosion and Materials Engineering, Gdansk University of Technology G. Narutowicza 11/12 80-233 Gdansk Poland

## Abstract

The effect of hexadecyltrimethylammonium bromide (CTAB) addition on the crystal structure, morphology, and magnetic properties of co-precipitated hexagonal barium ferrite was investigated. For a fixed amount of surfactant, different Fe^3+^ concentrations and Fe^3+^/Ba^2+^ ratios were used to optimize the formation of single-phase barium ferrite particles. The results indicated that the obtained ferrite particles exhibited coercivity changes similar to those of superparamagnetic particles with larger than theoretically calculated particle sizes. This results from the softening of the material due to the size reduction of the grains and incorporation of excess barium, localized on the surface of the particles. Therefore, lowering the energy barrier required to reverse the magnetization was observed, while high magnetization saturation was preserved. The precipitation of barium ferrite particles from a surfactant-rich solution allowed control of BaFe_12_O_19_ magnetic properties without introducing any modifications inside the crystal structure.

## Abstract 2

Observed morphology evolution of BaFe_12_O_19_ ferrite particles, obtained with different relative amounts of surfactant used.

## Graphical abstract

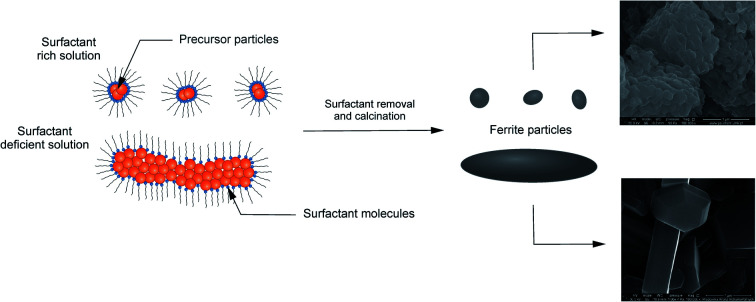

## Introduction

The importance of magnetic compounds has significantly grown in the past years as their new applications are being intensively developed in the fields of medicine,^1–3^ separation technology,^4–7^ preparation of smart materials^8,9^ and electronics.^10,11^ This results in an ongoing challenge to design new materials with the desired properties for a particular application or to develop new methods of their preparation. For the commonly studied ferrite ceramics, their magnetic properties result from interactions between metal ions occupying suitable positions relative to oxygen ions in their crystalline structure.^12–14^ In this regard, ferrites with hexagonal symmetry are an important class of materials with unique magnetic properties, such as high values of coercivity, magnetization, exchange stiffness, and strong magnetic anisotropy.^15^ Recently, their application in photocatalysis,^16–18^ water treatment processes,^19^ and hyperthermia application^20,21^ was studied. The most commonly used methods of hexagonal ferrite particles preparation are ball milling,^22,23^ thermal treatment,^24,25^ hydrothermal treatment,^26,27^ sol–gel autocombustion^28–31^ and chemical co-precipitation method.^32–36^ At the same time other works also suggest that simple preparation of hexagonal-based magnetic compounds is possible from iron-rich industrial wastes.^37^

Compared to spinel ferrites, materials like BaFe_12_O_19_ (BaM), being M-type hexagonal ferrite, are usually prone to higher magnetization and exhibit strong uniaxial magnetic anisotropy. The magnetic properties of BaM materials are associated with the changes in the microstructure and ions substitution. Despite the great potential of hexaferrites, methods of their synthesis and possible control over morphology and final magnetic properties, which are crucial considering their application, are still far less investigated compared to spinel analogues (*e.g.*, ZnFe_2_O_4_, Fe_3_O_4_). Most of the recent work in this field is focused on doping of the ferrite structure with transition metals and rare earth elements,^38–46^ while little attention is given to morphological and size-dependent evolution of BaM's properties.

In this regard, the presented study focused on the preparation of BaFe_12_O_19_ by co-precipitation of Fe^3+^ and Ba^2+^ ions in the presence of a cationic surfactant (CTAB). At present, CTAB addition was found to influence slightly on the properties of precipitated hexaferrites, however, its' effect was studied only at a limited range of introduced substrates.^32,33,47^ Therefore, in the presented study correlation of BaFe_12_O_19_ crystal structure with reagents concentration and reaction dynamics was investigated for the first time. The structural, textural, and surface characteristics' were performed to understand the structural evolution of the barium ferrite particles. The physical properties measurement system (PPMS) at the temperature of 293 K and in the range of 0–3 T was used to investigate the magnetic properties change as a function of mean particle size and the presence of BaFe_2_O_4_ and α-Fe_2_O_3_ impurities in the structure of barium hexaferrite.

## Experimental

### Preparation of barium hexaferrite particles

All the reagents were of analytical grade, purchased from Sigma-Aldrich (Poznan, Poland). In order to obtain BaFe_12_O_19_ particles, corresponding amounts of Fe(NO_3_)_3_·9H_2_O and Ba(NO_3_)_2_ were dissolved in distilled water, and 1000 mg dm^−3^ of hexadecyltrimethylammonium bromide (CTAB) was added. The aqueous solution of metal ions stabilized with the addition of a cationic surfactant was mixed during precipitation reaction. The precipitate was obtained by adding a fresh-made 5 M NaOH solution to pH value above 11 under room conditions. Obtained powders were centrifuged, washed with distilled water, dried at 80 °C, ground and then calcined in two steps: with a heating rate of 3 °C min^−1^ to the temperature of 180 °C for 45 minutes, then with a heating rate of 10 °C min^−1^ to 1000 °C for 2 h.

In contrast to previously reported in the literature,^32,33,36,48–50^ NaOH was added rapidly to solution at a rate of 15 cm^3^ s^−1^. In order to study the effect of reaction dynamic, two control samples with different Fe^3+^/Ba^2+^ ratios were prepared by adding NaOH dropwise at a rate of 0.03 cm^3^ s^−1^. Moreover, the influence of the precipitating agent was also investigated. In this regard, NH_4_OH/(NH_4_)_2_CO_3_ mixture was used as precipitant, where OH^−^/CO_3_^2−^ molar ratio was equalled to 2 : 1. All the obtained barium hexaferrite nanoparticles (BaM) are listed in Table 1 and labelled using two numbers, first one indicating Fe^3+^/Ba^2+^ ratio and a second one setting them following the rising concentration within the series. Samples prepared by dropwise precipitation or using carbonate/hydroxide mixture as a precipitating agent are additionally marked with “D” or “C” letters, respectively.

**Table 1:** Preparation conditions and physicochemical characteristics of the obtained barium hexaferrite particles

Fe^3+^/Ba^2+^	Sample	*C*_Fe^3+^_ [mol dm^−3^]	NaOH addition tempo [cm^3^ s^−1^]	Impurities [weight%]		*M*_S_ [Am^2^ kg^−1^]	*M*_R_ [Am^2^ kg^−1^]	*M*_R_/*M*_S_	*H*_C_ [kA m^−1^]	BET [m g^−1^]
				α-Fe_2_O_3_	BaFe_2_O_4_					
8	BaM_8_1	0.00825	15	—	—	64	17	0.27	14.1	1.8626
	BaM_8_2	0.05	15	—	13%	65	20	0.31	7.1	1.2487
	BaM_8_3	0.1	15	—	38%	57	17	0.3	26.6	1.4735
	BaM_8_3D	0.1	0.033	—	33%	64	16	0.25	10.8	0.7274
10	BaM_10_1	0.00825	15	—	—	69	11	0.16	6.0	4.7213
	BaM_10_1D	0.00825	0.033	6%	—	66	24	0.36	27.7	—
	BaM_10_1C	0.00825	15, CO_3_	—	—	—	—	—	—	8.5884
	BaM_10_2	0.1	15	—	6%	48	8	0.17	4.6	1.2588
	BaM_10_3	0.25	15	—	17%	58	16	0.28	23.3	1.0783
	BaM_10_4	0.33	15	—	21%	59	24	0.41	120.3	0.4541
12	BaM_12_1	0.00825	15	30%	—	48	23	0.48	287.5	0.3355
	BaM_12_2	0.1	15	—	—	67	30	0.45	125.2	0.875
	BaM_12_3	0.25	15	—	—	57	27	0.47	215.7	2.7436
	BaM_12_4	0.33	15	—	—	58	27	0.46	208.5	3.6066
	BaM_12_4C	0.33	15, CO_3_	4%	—	—	—	—	—	3.3672

### Physicochemical characterization

The crystal structure of the samples was determined by XRD analysis, performed using Rigaku Intelligent X-ray diffraction system SmartLab, equipped with a sealed tube X-ray generator. The scan rate was 1 °·min^−1^ with a step of 0.01° and in the range of 2*θ* from 15° to 75°. Qualitative analysis was performed using an external standard RIR method based on the ICDD database.

Morphology and specific surface area of the obtained samples were analysed using Quanta 250 FEG scanning electron microscope and Brunauer–Emmett–Teller (BET) isotherm method, recorded by measuring nitrogen adsorption at the temperature of liquid nitrogen using Micrometrics Gemini V, apparat model 2365.

The surface composition was examined by X-ray photoelectron spectroscopy (XPS) with energy-dispersive X-ray spectroscopy. In this regard, the barium ferrite particles were placed on carbon tape in a copper holder and dried under vacuum. The XPS spectra were recorded on Escalab 250Xi (Thermofisher Scientific using Mg K X-rays). Elemental composition was examined by wavelength dispersive X-ray fluorescence spectroscopy, recorded using Tiger S8 spectrometer (Bruker).

Magnetic properties were determined by analysing hysteresis loops using Physical Properties Measurement System (PPMS) (Quantum Design, San Diego, CA, USA) at the temperature of 293 K and in the range of 0–3 T.

## Results and discussion

### Crystal structure

**Fig. 1:**
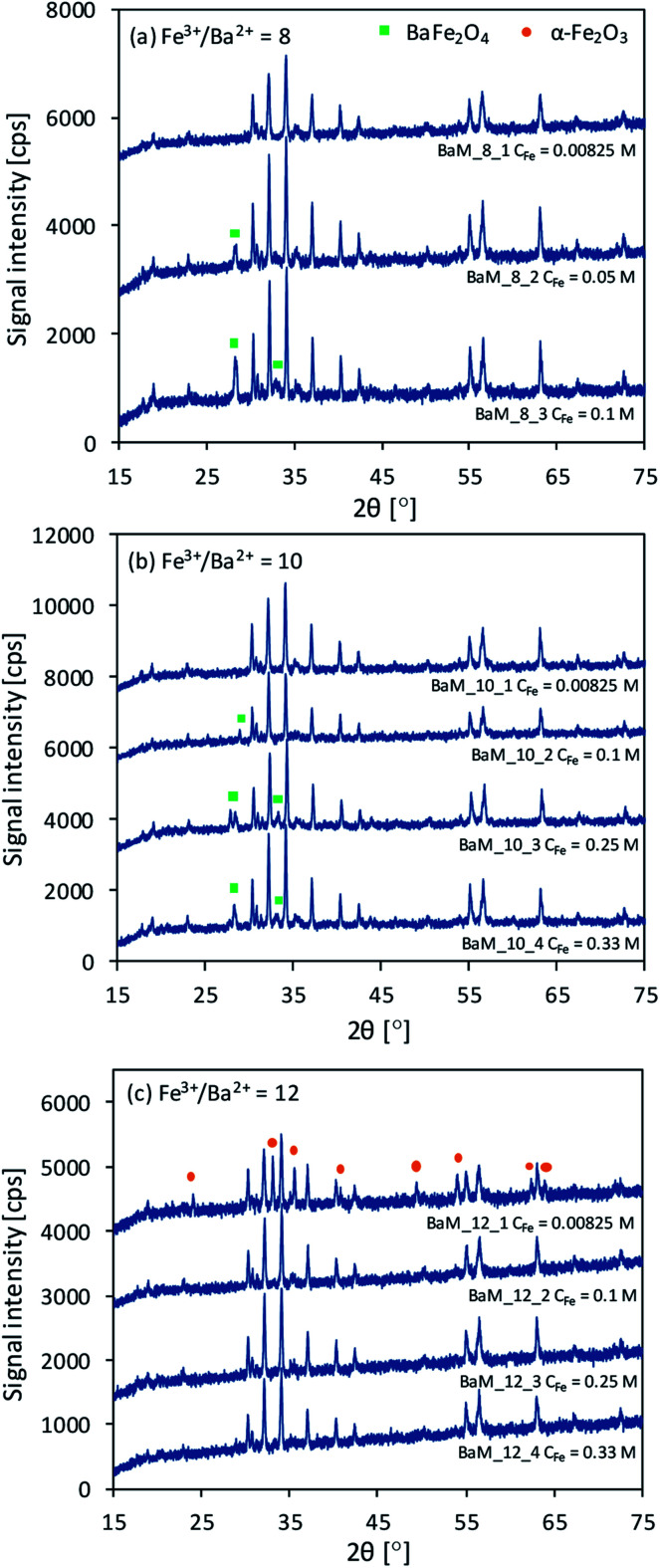
XRD patterns for the obtained BaM samples. Not indexed peaks corresponds to BaFe_12_O_19_ phase.

The XRD results are presented in Fig. 1a–c, while calculated impurities presence are presented in Table 1. The crystallite sizes of barium hexaferrite were in the range of 45 to 55 nm, according to Sherrer's equation. For all the obtained samples, the formation of the BaFe_12_O_19_ (BaM) structure was observed. However, changing the concentration of ions resulted in the formation of additional phases of barium monoferrite BaFe_2_O_4_ and α-Fe_2_O_3_, as shown on the XRD patterns in Fig. 1. These impurities commonly occur after the non-stoichiometric precipitation of BaM. Observed signals of BaFe_12_O_19_ and α-Fe_2_O_3_ correspond to ICDD's card numbers 9008137 and 2101167, respectively, while for BaFe_2_O_4_ signals corresponds mostly to card no. 4107896 (space group *Bb*2_1_*m* with the main signal at 28.46°). However, left-shifted main peak at 27.9° for sample BaM_10_3 was observed, which suggested the formation of other BaFe_2_O_4_ structures according to card no. 2002358 (space group *Pmcn* with the main signal at 27.97°). Throughout the results not indexed peaks corresponds to BaFe_12_O_19_ only.

It was found that the appropriate molar ratio of Fe^3+^ to Ba^2+^ ions and their concentration determine the purity of the obtained BaFe_12_O_19_ particles. The samples obtained from concentrated solution (*C*_Fe_ > 0.00825 M) with the molar ratio of Fe^3+^ to Ba^2+^ equalled to 8 and 10 were characterized by the presence of BaFe_12_O_19_ and BaFe_2_O_4_ phases. Moreover, the BaM signal intensity for the samples was generally higher, comparing to pure samples from these series. The formation of the hexagonal phase occurs through the solid-state reaction between BaFe_2_O_4_ and α-Fe_2_O_3_,^51^ during which BaFe_2_O_4_ particles should be surrounded by predominant α-Fe_2_O_3_. Therefore, an increase in Ba^2+^ ions concentration in the precipitate can facilitate the formation of BaM, as the mean diffusion length of Ba^2+^ ions needed to complete the reaction shortened. Finally, high crystallinity particles could be easily formed in the presence of BaFe_2_O_4_ excess. On the other hand, the increase of the concentration inside Fe^3+^/Ba^2+^ = 12 series could not result in non-stoichiometric barium excess. Therefore suppression of BaFe_12_O_19_ formation occurred, and due to the presence of BaFe_2_O_4_ and α-Fe_2_O_3_, additional calcination of samples BaM_12_3 and BaM_12_4 proceeded (XRD patterns before this are shown in ESI†).

**Fig. 2:**
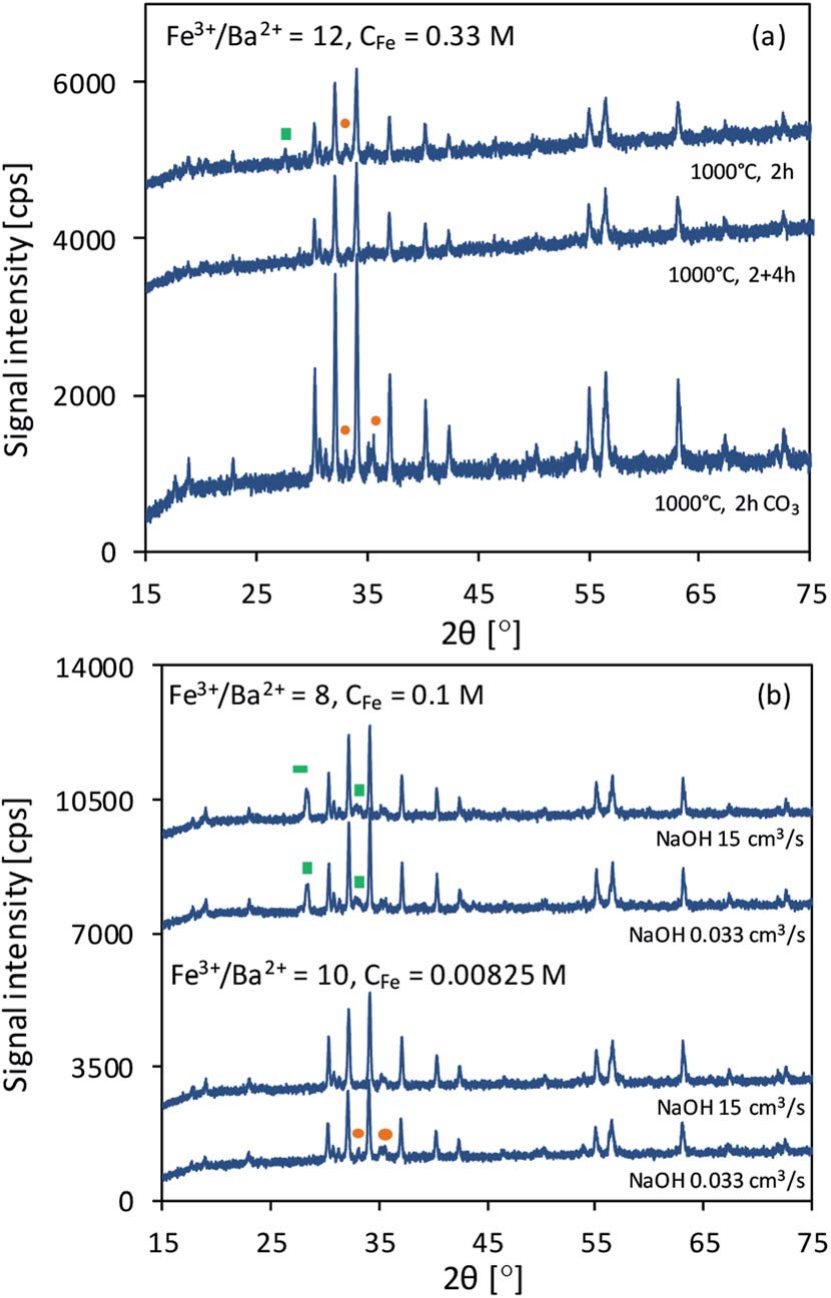
Difference in XRD patterns of samples obtained using OH^−^/CO_3_^2−^ as precipitating agent (a) and with different NaOH addition tempo (b).

To confirm the crucial effect of ions concentration, additional samples of barium hexaferrite (marked as “C” in Table 1) were precipitated with a mixture of OH^−^ and CO_3_^2−^ from solutions analogical to BaM_10_1 and BaM_12_4. Both of the samples were found to be pure or almost pure BaFe_12_O_19_. Moreover, as shown in Fig. 2b, sample BaM_12_4C was much more crystalline than BaM_12_4, due to the direct formation of BaCO_3_ instead of Ba(OH)_2_, which could affect the distribution of Ba^2+^ ions inside the precipitate.

As seen in Fig. 2b, altering the rate of precipitation from 15 cm^3^ s^−1^ to 0.033 cm^3^ s^−1^ resulted in a lower content of BaFe_2_O_4_ phase (see samples BaM_8_3 and BaM_8_3D with 38% and 33% of BaFe_2_O_4_ by weight according to the results of RIR analysis) and formation of α-Fe_2_O_3_ (see samples BaM_10_1 and BaM_10_1D).

### Morphology and surface characterization

As presented in Table 1, the BET surface area varied from 0.3 m^2^ g^−1^ to 8.6 m^2^ g^−1^ for BaM_12_1 and BaM_10_1C, respectively. The observed development of the specific surface area for samples within series 10 and barium hexaferrite particles precipitated with the mixture of CO_3_^2−^/OH^−^ may result from the stabilization of precipitated particles and inhibition of their secondary growth. The presence of carbonates in the precipitation environment affected the morphology of the final particles. The introduction of carbonates increased the content of BaCO_3_/Fe_2_(CO_3_)_3_ in the precipitate, which may result in a relatively higher surface area.

**Fig. 3:**
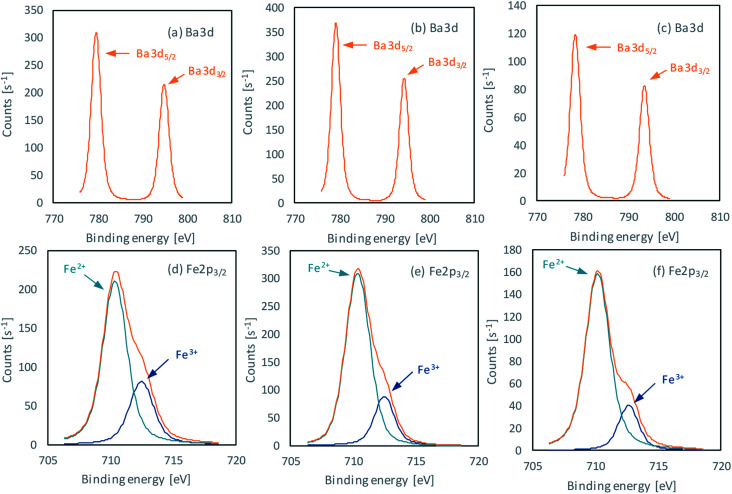
XPS signals of Ba and Fe elements, obtained for pure BaFe_12_O_19_ samples BaM_10_1 (a and d), BaM_12_2 (b and e) and BaM_12_3 (c and f).

The main XPS results for the selected, pure BaFe_12_O_19_ samples with different Fe^3+^/Ba^2+^ ratios are illustrated in Fig. 3 and listed in Table 2. After subtracting baseline, in which C 1s peak at 285 eV was used for charge correction, the peaks at ∼795 eV and ∼779 eV are ascribed to Ba 3d_3/2_ and Ba 3d_5/2_ peaks, respectively. The XPS spectrum of Fe 2p can be resolved in two peaks, which are ascribed to Fe^2+^ at ∼710 eV and Fe^3+^ at ∼712 eV. For all samples, the percentage amount of Fe^2+^ was higher than Fe^3+^ ions at the BaFe_12_O_19_ surface, which could result from oxygen vacancy inside the structure, which caused Fe^3+^ reduction to compensate charge distribution.^52,53^ The highest content of Fe^2+^ compared to Fe^3+^ was observed for the sample BaM_12_3, which can contribute to the lower *M*_S_ value of this sample. However, for all samples, Fe^3+^ content was quite low, comparing to other reported results (approx. 72% of Fe^2+^ in this study *vs.* 27% in ref.54), suggesting that surface composition plays a minor role in overall sample magnetization.

**Table 2:** Binding energy of analyzed elements and calculated energy difference between observed signals

Sample	C 1s [eV]	Ba 3d_5/2_ [eV]	Fe 2p_3/2_ [eV]	O 1s [eV]	ΔBa-O [eV]	ΔFe-O [eV]	Ref.
BaM_10_1	284.18	780.08	710.64	529.78	250.30	180.86	This study
BaM_12_2	184.16	779.15	710.38	529.75	249.40	180.63	
BaM_12_3	184.16	778.43	710.20	528.85	249.58	181.35	
BaFe_12_O_19_	284.80	779.30	710.40	529.60	249.70	180.80	55
Fe_2_O_3_	—	—	711.20	530.20	—	181.00 min	56,57
	—	—	—	529.50	—		
BaCO_3_	—	779.30	—	530.80	248.60 max	—	58–60
	—	779.40	—	531.00		—	
Ba(OH)_2_	—	779.20	—	530.30	248.90	—	59,60

Moreover, the presence of Ba^2+^ intermediates such as BaCO_3_, and Ba(OH)_2_ at the surface of obtained BaM was analysed based on the energy difference between observed metal–oxygen signals. The ΔBa-O differences were higher than 248.9 eV for all samples, which is characteristic for BaM. For BaM_10_1 and BaM_12_2 their overall spectra were similar to those obtained by Atuchin *et al.* in their study on BaFe_12_O_19_ electronic structure.^55^ For sample BaM_12_3 no other barium compounds were observed on the surface of BaFe_12_O_19_. However, its surface was visibly enriched in iron, which was followed by a higher ΔFe-O energy difference. It suggests that the BaM_12_3 surface structure was more similar to iron oxide rather than barium ferrite.^58–60^ The presence of amorphous, iron-rich structure could additionally explain the decrease of measured magnetization.

**Fig. 4:**
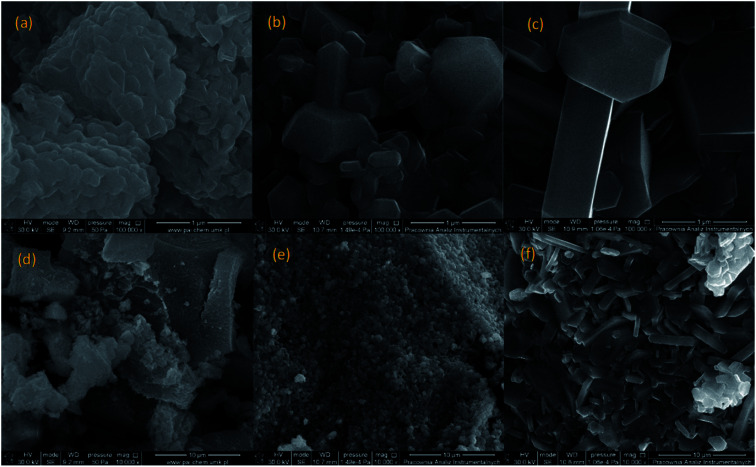
SEM images of the obtained single phase BaFe_12_O_19_ samples: BaM_10_1 (a and d), BaM_12_2 (b and e) and BaM_12_3 (c and f).

The morphology of BaFe_12_O_19_ was studied by SEM, and exemplary images of the selected samples BaM_10_1, BaM_12_2, and BaM_12_3 are presented in Fig. 4 (all were found to be single-phase BaFe_12_O_19_). As expected, the changes in the reaction conditions resulted in clearly visible microstructure differences of the obtained materials. For sample BaM_10_1 formation of ultrafine, irregular-shaped grains sintered into larger aggregates were observed. For both samples BaM_12_2 and BaM_12_3, the grains formed regular hexagonal platelets or bifrustums crystals. Among the samples, BaM_12_3 ferrite particles had a larger grain size, which could be a direct result of the increased substrates concentration and additional sintering required to form the single-phase BaFe_12_O_19_. For a detailed study of obtained particles, the distribution of BaM grains' width and height of at least 200 grains for each sample is presented in Fig. 7. An increase of the CTAB/Fe^3+^ ratio resulted in both decreasing grains' size and narrowing the size distribution. Since the introduced CTAB at the preparation step should be removed at approx. 400–500 °C during the sintering,^61^ its presence is mostly assumed to affect precipitation process, as the BaFe_12_O_19_ nucleation was found to start only in the range of 600 to 700 °C (see ESI for XRD patterns†). Therefore, observed differences are expected to result from the evolution of precipitated, amorphous precursor. In general, surfactant presence influence the formation of primary particles and their secondary growth, *e.g.*, through their stabilization and inhibition of Ostwald ripening.^27,62^ Since the same process can be discussed for amorphous precipitates,^63^ a similar stabilization is expected to take place inside the investigated system. The effectiveness of such a process should depend on the CTAB amount that adsorbed on the particles surface. Therefore increase in the Fe^3+^ and Ba^2+^ concentration, followed by the formation of larger amounts of the precipitate, could result in not effective inhibition of the particle growth in the presence of CTAB. The BET analysis of precipitated precursor confirmed the changes for samples BaM_10_1 and BaM_12_4 with the specific surface area of 124 and 36 m^2^ g^−1^, respectively.

**Fig. 5:**
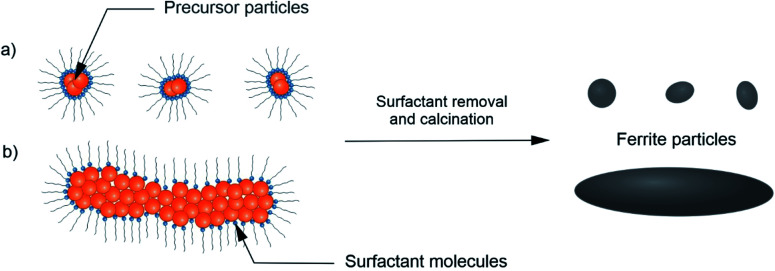
Scheme of the possible CTAB effect on the observed BaFe_12_O_19_ size evolution during synthesis from diluted solution (a) and concentrated solution (b) of Fe^3+^ and Ba^2+^.

Therefore, the change in CTAB/Fe^3+^ ratio is responsible for the size evolution of the obtained precursor, and is further reflected in the observed grains size of the final ferrite particles. An increase of the precursors' size could also influence on the longer calcination time of sample BaM_12_4, which could additionally intensify observed grains growth. Discussed mechanism is presented schematically in Fig. 5. A crucial role of surfactant on the grains size was also confirmed by additional SEM analysis of sample BaM_10_4, which was found to form polydisperse mixture of platelet particles, mostly in the range of 1 to 20 micrometres size (see ESI for exemplary images†).

### Elemental analysis

**Fig. 6:**
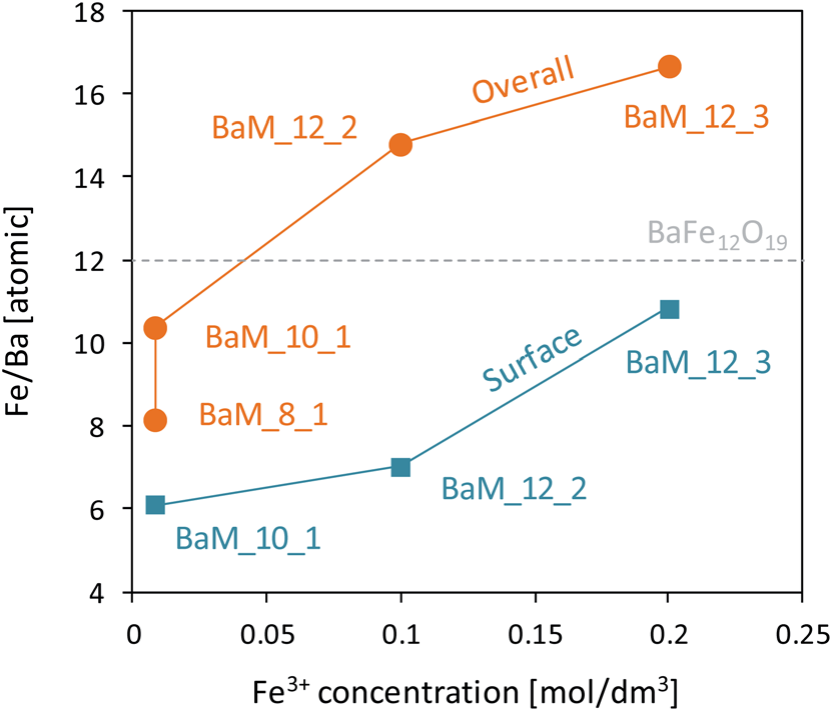
The ratio of Fe/Ba observed in XRF spectra for synthesized single-phase BaFe_12_O_19_ and its correlation with the surface composition revealed by XPS analysis.

The XRF results showed relative amounts of Fe and Ba for the selected samples, see in Fig. 6. All the samples were single phase of BaFe_12_O_19_. It was shown that the elemental composition followed a similar trend as observed in XRD patterns, and a significant differences were noticed between the samples despite their single-phase character. Samples synthesized in barium rich environment (Fe^3+^/Ba^2+^ = 10 or 8) exhibited visible barium excess, while samples prepared at stoichiometric conditions were enriched in iron. Material's non-stoichiometry is a known factor that could affect the magnetic properties of M-type hexaferrites. It could result from changes in the crystalline structure and Fe^3+^ site occupancy, which is critical for ferrite materials. Prathap *et al.* and Zhao *et al.* have both studied the effect of iron non-stoichiometry in terms of its deficiency and surplus, respectively.^64,65^ Their results showed that in general, both magnetization and coercivity of the material should be proportional to iron content. On the other hand, XPS analysis revealed non-stoichiometry of the surface composition, and the general trend was proportional to the bulk composition from XRF, as shown in Fig. 6. Moreover, despite the overall iron excess/barium insufficiency of samples BaM_12_2 and BaM_12_3 the surface was still enriched in barium. It is consistent with the observation of Atuchin *et al.* and shows that Ba tends to locate on the surface of BaFe_12_O_19_ phase.^55^ It could be important for samples created in barium rich environment as suggest that overall Ba excess could tend to localize on the grains surface and possible their boundary inside the material.

**Fig. 7:**
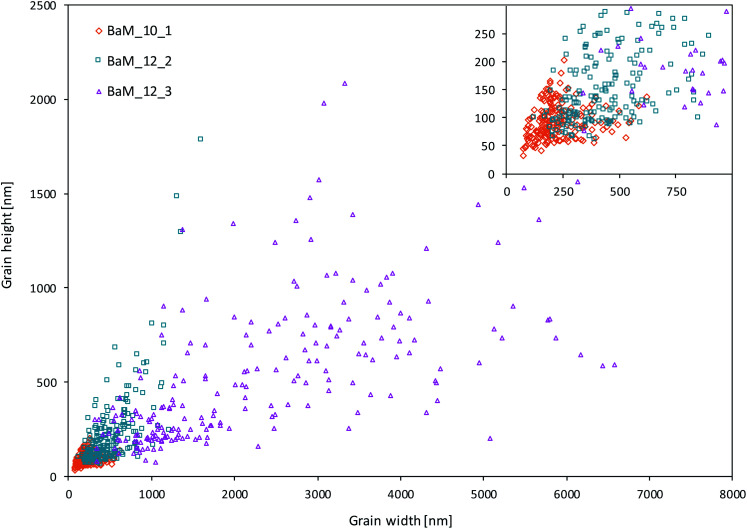
Size distribution of grains observed by SEM analysis.

### Magnetic properties and microstructure

**Fig. 8:**
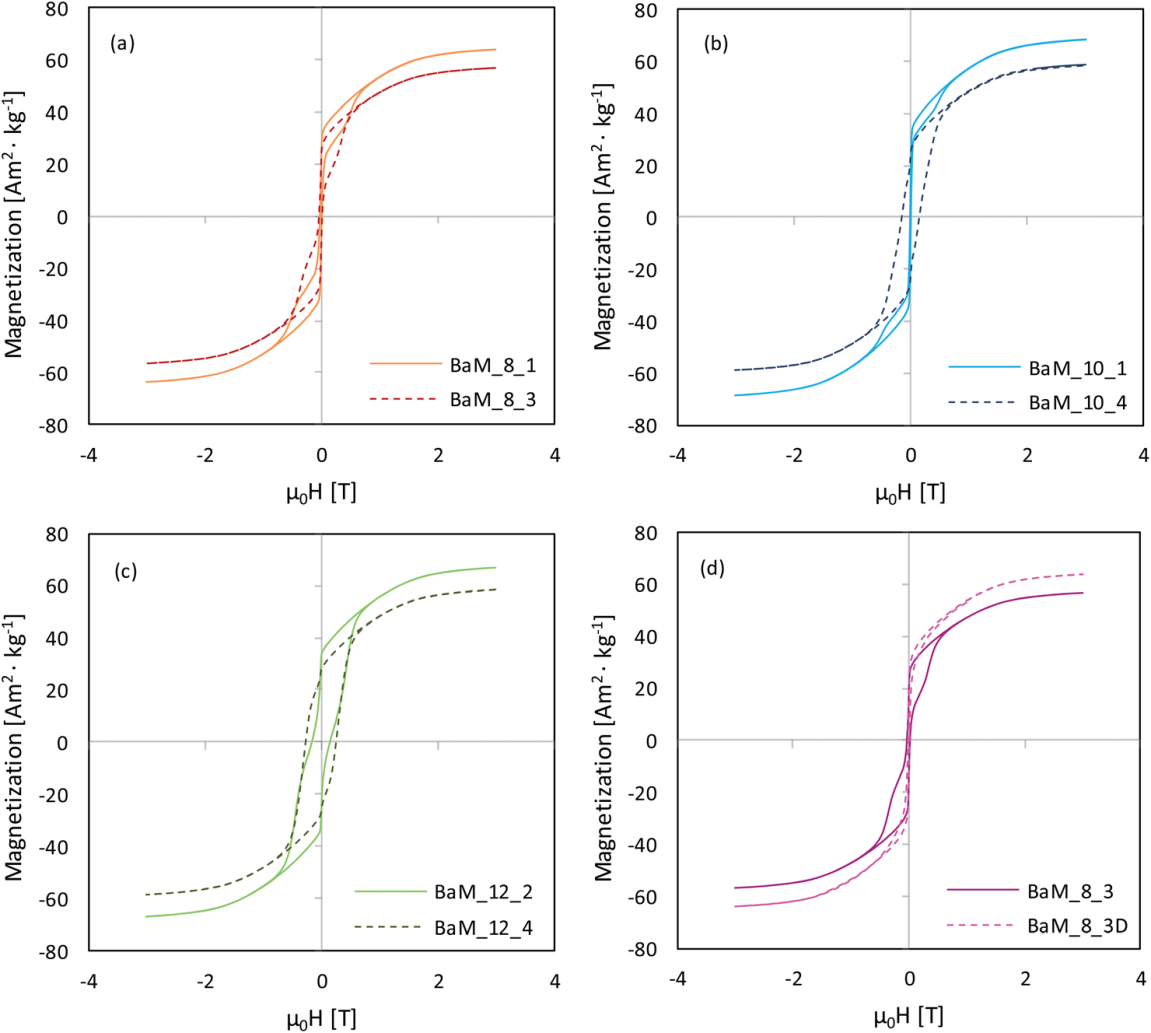
Hysteresis loops of the selected BaFe_12_O_19_ samples obtained within Fe^3+^/Ba^2+^ series of 8 (a), 10 (b) and 12 (c), together with the difference between fast and slowly precipitated samples (d).

The PPMS analysis results are presented in Table 1, while obtained hysteresis loops for the most different samples in each series are shown in Fig. 8a–d. The sample BaM_12_1, due to the high content of paramagnetic α-Fe_2_O_3_ was excluded from general comparison, and its magnetic properties were discussed further. In general, the magnetization saturation (*M*_S_) values for obtained BaM were quite similar and ranged from 58 to 68 Am^2^ kg^−1^ for samples BaM_12_4 and BaM_10_1, respectively. For some samples, obtained values were very close to the theoretical value of 72 Am^2^ kg^−1^.^15^ In all series, the highest magnetization was observed for the single-phase BaFe_12_O_19_ samples, since both BaFe_2_O_4_ and α-Fe_2_O_3_, occurring as impurities, possess no or weak ferromagnetic properties.

**Fig. 9:**
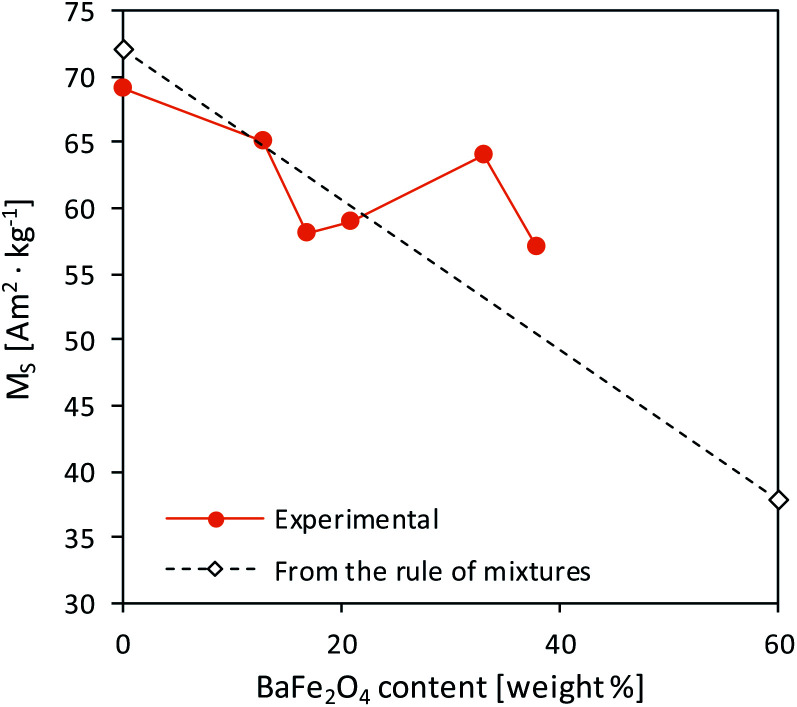
Theoretical and experimental dependence between *M*_S_ and BaFe_2_O_4_ content.

However, *M*_S_ value did not decrease linearly with increasing BaFe_2_O_4_ content, suggesting that there were synergic interactions between both barium phases. As presented in Fig. 9, the highest divergence between theoretical and experimental *M*_S_ values was observed for samples possessing approx. 33% of BaFe_2_O_4_ by weight. Previously, Pahwa *et al.* reported the enhanced magnetization in the BaFe_12_O_19_/NiFe_2_O_4_ system for the sample containing 30% of a NiFe_2_O_4_ spinel phase.^66^ BaFe_2_O_4_ saturation magnetization value was adapted as 15 Am^2^ kg^−1^, following the results by Javidan *et al.*^67^

For pure BaFe_12_O_19_ samples, *M*_S_ values were high except for samples BaM_12_3 and BaM_12_4, which needed additional thermal treatment. It was in agreement with XRD results, as they have shown suppression of BaFe_12_O_19_ formation in the samples, as mentioned above. Presented magnetic measurements results implied that approximately 20% of their content was not prone to magnetization, probably due to the creation of an amorphous phase, right next to the highly crystalline region. For other BaM samples, the paramagnetic phase was about 4–6%, which could be a net result of both amorphous content presence, spin canting on the surface of the material, and the iron deficiency confirmed by XRF analyses. In most of the presented hysteresis, there were also visible steps, which indicated that obtained samples did not behave uniformly.^66^ Because this phenomenon also applied to pure BaM samples, it could result from their polydispersity and interparticle interactions rather than differences in crystal structure itself.^68,69^ A smooth hysteresis was obtained by lowering the precipitation rate, as shown for sample BaM_8_3D in Fig. 8d.

**Fig. 10:**
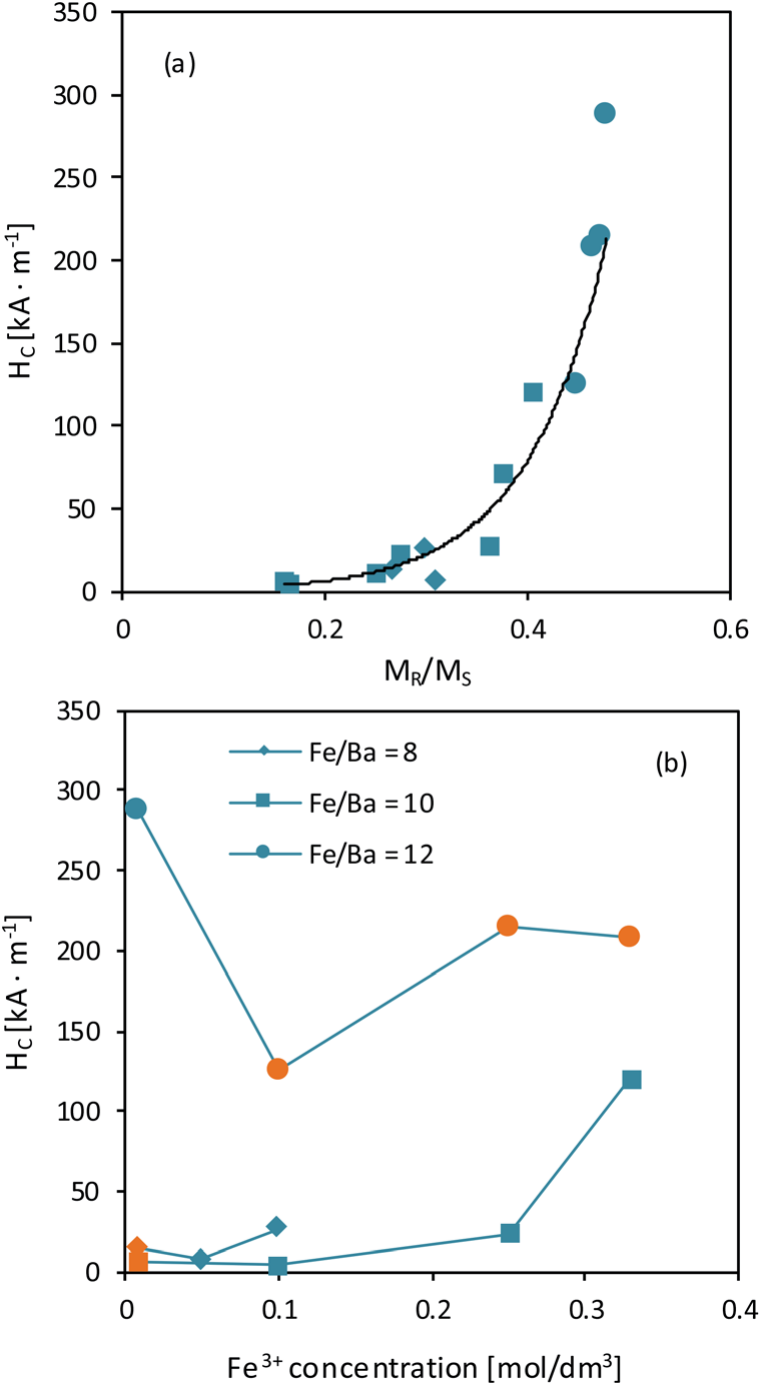
Correlation between *M*_R_ and *H*_C_ for the obtained BaM samples (a) and *H*_C_ variance with applied Fe^3+^ concentration for main samples in each series (orange points indicate pure BaFe_12_O_19_) (b).

Finally, the most noticeable changes were in magnetic coercivity (*H*_C_) and remanence (*M*_R_) of the obtained samples, between which a direct connection was found, as shown in Fig. 10a. Remanence values were normalized with *M*_S_ to exclude the effect of non-magnetic phases. In literature, BaFe_12_O_19_ coercivity usually fit between 200–500 kA m^−1^, while it is seen that most of the presented samples had significantly lower *H*_C_, ultimately reaching the difference between 6 and 215 kA m^−1^ (samples BaM_10_1 and BaM_12_3, respectively, both being single-phase BaM). The overall trend of *H*_C_ growth together with applied Fe^3+^ concentration and higher Fe^3+^/Ba^2+^ ratio was quite well observed and is shown in Fig. 10b. The only sample that was not fitting this relation was BaM_12_1, mostly due to approximately 30% of α-Fe_2_O_3_ in its' structure, which, compared to BaFe_2_O_4_, is strictly paramagnetic. The obtained results are in agreement with the literature showing that the presence of the non-magnetic layer between misoriented nano-grains/particles resulted in the enhancement of coercivity.^70,71^ The analogical statement could be made for samples 12.3 and 12.4, considering their high amorphous/non-magnetic phase content. The simultaneous decrease of both *M*_R_ and *H*_C_ suggested that material could tend toward its superparamagnetic state.^72^

It is well known that both coercivity and magnetic remanence depend on particle size, exhibiting maximum approximately at the point of single to multidomain transition.^73,74^ It results from magnetic domain motion as well as magnetization vector's coherent rotation for particles larger and smaller than a critical value, respectively. For BaFe_12_O_19_, this point is not strictly defined. However, as previously reported, it is between 500–1000 nm.^15^ Following this, most of the grains observed for BaM_10_1 and BaM_12_2 should behave as single-domain particles, while sample BaM_12_3 could be seen as multidomain. Therefore coercivity of BaM_12_3 should depend mostly on wall motion, that especially will become pinned at grain boundaries as reported by Dho *et al.*^25^ On the other hand, for a single domain particles, the energy barrier preventing magnetization reversal is proportional to magnetic anisotropy, following the relation Δ*E* = *KV*, where *K* is an anisotropy constant and *V* is particle's volume.

The superparamagnetic state is observed when this barrier can be overcome by thermal fluctuations, leading to spontaneous switching of the magnetization vector. For the obtained samples, an effective anisotropy constant was found by analysing the high-field region of hysteresis loops. In this region, magnetization changes reversibly due to the alignment of magnetic moments within the material with a magnetic field vector. Therefore, it causes them to drift away from their randomly-distributed easy magnetization axes. According to the law of approach saturation to magnetization (LAMS) this region could be described by equation:^75,76^
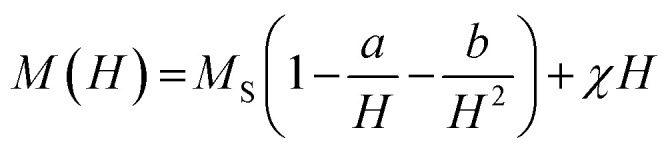
where *M* is magnetization, *H* is magnetic field strength, *χ* is high field magnetic susceptibility and *a* and *b* are numerical parameters corresponding to materials defects and anisotropy, respectively. While the direct interpretation of *a* is not well known, for a compound with hexagonal symmetry *b* is found to be:^77^
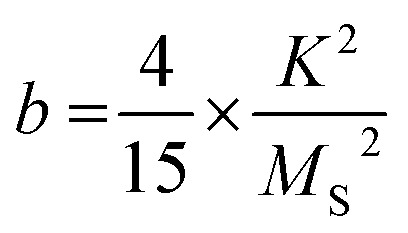
from which, effective anisotropy constant (*K*) could be easily calculated. Approximated values of best-fitting *a* and *b* parameters, together with obtained *K* values for selected samples are presented in Table 3.

**Table 3:** Best-fitting LAMS parameters and found K values for selected BaM samples

Sample	*a*[*T*]	*b*[*T*^2^]	*K* [J m^−3^]	*χ* [A T^−1^ m^−1^]
BaM_10_1	0	0.1940	3.10 × 10^5^	2613
BaM_12_2	3.66 × 10^−7^	0.2080	3.13 × 10^5^	2984
BaM_12_3	1.9 × 10^−3^	0.2141	2.70 × 10^5^	2483

**Fig. 11:**
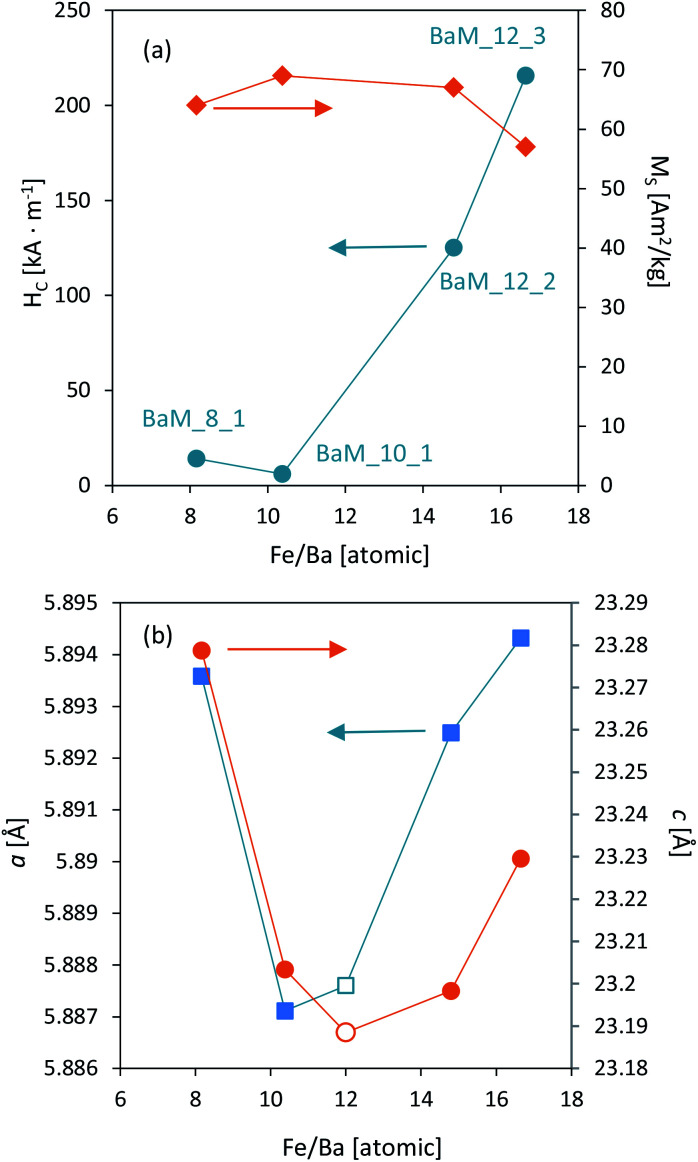
Correlation between observed XRF composition of the obtained single-phase BaFe_12_O_19_ samples and their magnetic properties (a) and structural parameters (b). Empty points indicate known value for BaM.

For samples BaM_10_1 and BaM_12_2 calculated *K* values were very close to a known value of 3.3 × 10^5^ J m^−3^,^15^ and the parameter *a* was 0 or had an extremely low value. As expected for BaFe_12_O_19_, calculated anisotropy values were high, and sizes of grains observed by SEM analysis were significantly larger than theoretically calculated for superparamagnetic particles. On the other hand, an alternative explanation of coercivity loss through possible wall motion, similar to sample BaM_12_3, could not be fully proven. In this case, grain growth should result in a smaller area of grain boundary and further *H*_C_ decrease through inhibition of wall pinning,^25^ which was not observed. It shows that other factors, outside of simple size reduction of BaM grains, are responsible for enhanced coercivity decrease. Observed non-stoichiometry of obtained samples could be an influencing factor on the overall magnetic properties of BaFe_12_O_19_, and the hypothetical barium/iron excess inside the ferrite structure is first to be considered. Prathap *et al.* have studied in detail the effect of iron deficiency on analogical lead hexaferrite's properties.^64^ Their results suggest that the formation of PbFe_12−*x*_O_19−*y*_ could indeed influence coercivity loss. However, it is accompanied by a significant loss in saturation magnetization value. Although a similar relation between *H*_C_ and measured Fe/Ba ratio could be observed in this study (see Fig. 11a), the *M*_S_ value appears to be independent of the material's composition. On the other hand, lowering of Fe/Ba ratio could be understood as the incorporation of barium surplus to the BaM lattice. Zhao *et al.* studied the effect of Ba surplus on the properties of BaCoTiFe_10_O_19_.^78^ Despite the natural differences in coercivity between BaM and its Co + Ti modified analog (being soft ferromagnetic), their results indicate that changing the ratio of (Fe + Co + Ti)/Ba from 12 to 10 should result in a significant *M*_S_ decrease and a unit cell extension. As shown in Fig. 11b, both *a* and *c* lattice parameters of sample BaM_10_1 are very close to a known value for BaFe_12_O_19_,^15^ indicated as empty points in Fig. 11b. However, possible barium incorporation could be observed for sample BaM_8_1 and is quite reasonable with increased barium content during the preparation. The possibility of Ba excess inside BaM structure for sample BaM_8_1 could also explain the decrease of its magnetization value, comparing to other samples. However, no visible increase in *H*_C_ was observed, that could have been expected.^78^

On the other hand, XPS analysis suggested that Ba tends to accumulate at the grains' surface. Composition change at the grain boundary can be an influencing factor on the coercivity of sintered material due to affecting the intergrain coupling and the properties of the boundary phase.^79,80^ Since no evidence of other crystalline-phases was observed based on the XRD patterns, the barium excess could be present as an amorphous, possibly thin layer on the grains' surface. Analogical layers were observed for modified Mn–Zn^81^ and SrFe_12_O_19_ (ref. 82) ferrites and were studied in detail for alloy magnets.^83,84^ The effect of such boundary-phase could vary significantly depending on its specific conditions and the morphology of the grains. Existing studies suggest that the coercivity of the hard magnetic phase could be significantly decreased if the distribution of the magnetic moments became misaligned at the boundary.^69^ By assuming that this misaligned region became enlarged by the existence of a non-stoichiometric surface phase, the overall material could be effectively softened. This reasoning could explain the observed drop of coercivity for materials obtained at barium rich conditions, as a mixed effect of size reduction and modification of boundary properties through the formation of the barium-rich layer. On the other hand, samples characterized by the iron excess are in agreement with the study by Zhao *et al.* focused on the formation of BaFe_12+*x*_O_19+1.5*x*_ ferrites.^65^ Their results suggest that incorporation of iron surplus inside BaM lattice both expands its unit cell and increase observed *M*_S_ and *H*_C_ values. In this study, both coercivity increase and unit cell enlargement were observed for samples BaM_12_2 and BaM_12_3, as shown in Fig. 11a and b. Moreover, the XPS analysis confirmed that no non-stoichiometric iron excess is not present at the surface. On the other hand, both samples possessed rather small values of *M*_S_, comparing to increased magnetization reported by Zhao and co-workers. However, it could be a result of structure deficiency, which was already revealed for sample BaM_12_3.

**Fig. 12:**
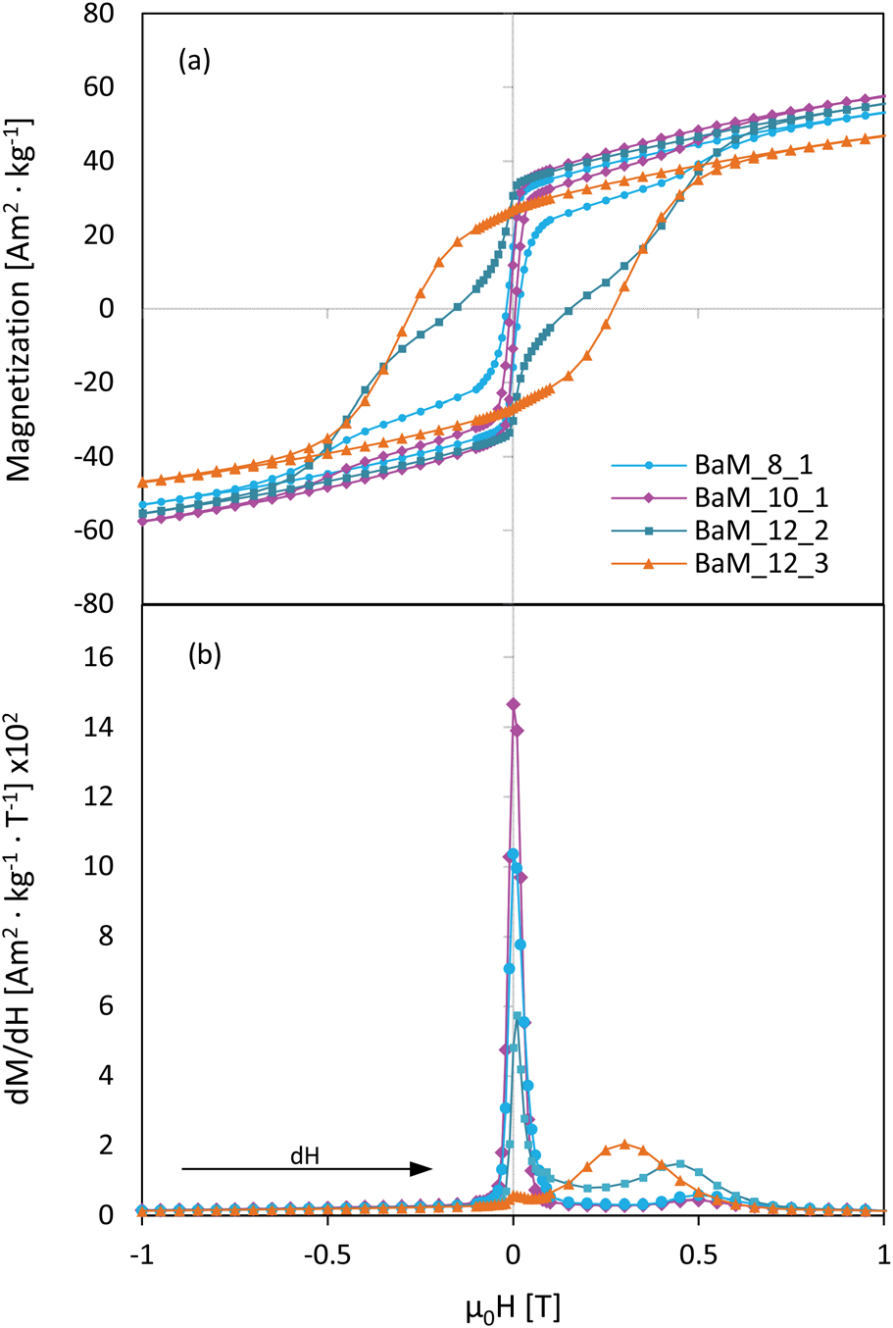
Parts of a magnetic hysteresis loops for selected BaFe_12_O_19_ samples (a), together with a corresponding differentiate d*M*/d*H* curves (b).

In order to gain a better insight into magnetic interactions occurring inside synthesized materials, a differential d*M*/d*H* curves were analysed for selected single-phase BaM samples. Obtained results are presented in Fig. 12b showing changes between III to I quadrants of the hysteresis, with a range limited from −1 to 1 T (outside this, no other peaks were observed). It was found that samples obtained at different conditions were characterized by different dM/d*H* character. Sample BaM_12_3 exhibits typical behaviour for hard ferromagnetics, with a single peak dominating in a differential curve at *H* ≈ 0.3 T and a smooth, broad *M*(*H*) hysteresis. On the other hand, sample BaM_12_2 possesses two visible peaks around *H* ≈ 0 and *H* ≈ 0.47 T, which is characteristic for a weakly coupled magnetic systems.^66,85^ It was changed for samples BaM_8_1 and BaM_10_1 for which the *H* ≈ 0.5 T peak disappears almost completely (especially for BaM_10_1). The systematic disappearance of the second peak on the d*M*/d*H* curve could indicate an enhancement of the coupling behaviour inside the material. For a BaFe_12_O_19_, coupling with a soft magnetic phase could lead to a coercivity loss.^86,87^ However, the final properties should heavily depend on the fraction of both phases. As throughout the Fe^3+^/Ba^2+^ = 8 and 10 series, no strong dependence between magnetic properties and synthesis conditions was observed, and therefore between possible Ba content, it seems unlikely that similar coupling is mostly responsible for observed *H*_C_ loss. Moreover, no enhancement of the remanence was observed, which should be characteristic for exchange-coupled BaM composites regardless of the simultaneous *H*_C_ change.^86–89^ Final remanence value was always proportional to the coercivity, as shown before, and the highest *H*_C_ was observed for sample BaM_10_4, which was also visibly less coupled than BaM_10_1 (see in the ESI for the comparison of the d*M*/d*H* curves†). On the other hand, the same sample also possessed visibly larger grains than BaM_10_1, suggesting that observed particles size was still significant for the final properties. Alternatively, dipolar interactions could also be responsible for observed hysteresis behaviour. In this regard, simultaneous *H*_C_ and *M*_R_ decrease was characteristic for more interacting particles.^90,91^

A rough estimation of the energy of such interactions can be made through relation:^92^
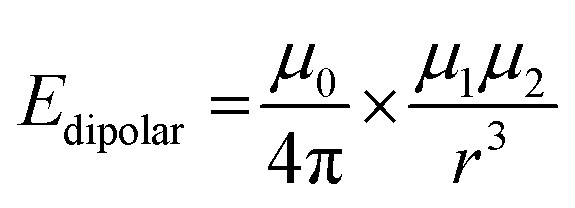
where *μ*_0_ is magnetic constant, *μ*_1_ and *μ*_2_ are magnetic moments of interacting particles (grains) and *r* is distance between them.

**Fig. 13:**
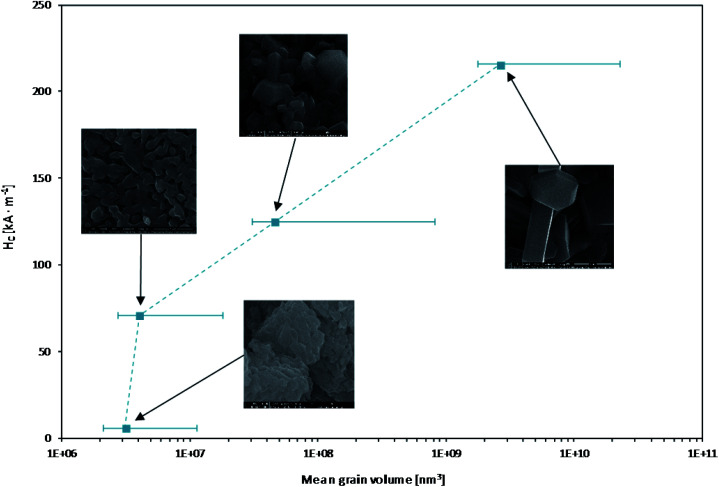
Coercivity dependence on grain volume for BaFe_12_O_19_ samples.

It can be further multiplied by the number of neighbouring particles (*n*) and for sample BaM_10_1 calculated energy indeed could be an important factor. Especially, considering relatively big particle interacting with a smaller one, anisotropy of the later one could be overcomed (for a 115 × 50 nm ellipsoid interacting with a 245 × 110 nm one, at the distance of 90 nm and with *n* = 5, calculated *E*_dipolar_/*E*_anizo_ = 1.073). Importance of the dipolar interactions can somehow explain differences observed between samples BaM_10_1 and its resynthesized version. The new sample was characterized with similar XRD, morphology, size distribution, BET, *M*_S_, and *K* values as the original one and a slightly higher *H*_C_ and *M*_R_, which could be connected to an increase in distance between the particles and the number of a possible neighbours. It was shown in Fig. 13, where obtained relationship between particles size and *H*_C_ was presented (*V* was calculated as a mean, weighted with a size distribution for every sample (Fig. 7), treated as ellipsoids of revolution).

The overall results showed that the observed coercivity decrease results mostly from the size reduction of BaM grains and possible increase in their interactions. Especially, dipolar interactions could be an important factor, considering ultrafine particles obtained in barium-rich conditions. It was followed by the change in elemental composition and particularly incorporation of barium surplus tends to localize on the boundary of the particles. This behaviour may further affect interactions between the grains and therefore resulting properties. For ultrafine particles, this could lead to a more collective state of a material, where magnetization reversal is a continuous process of succeeding switches of neighbouring grains rather than anisotropy overcoming for isolated particles. Ultimately, by changing the size and composition of obtained materials, they became effectively softened, mimicking the superparamagnetic behaviour, despite being far from their calculated critical size and remaining ferrimagnetic (none of it reached *H* = 0 and *M* = 0 points during measurements).

Previously, in the literature, the observed grains had similar dimensions and significantly larger *H*_C_ values.^25,62^ In this study, both phase and elemental composition of BaFe_12_O_19_ was found to vary depending on the applied Fe^3+^/Ba^2+^ ratio and the relative amount of surfactant. Therefore, both of these parameters are important for the preparation of a single-phase barium hexaferrite particles with different coercivity. Ultimately, significant changes in morphology and magnetic properties of BaM could be observed with a high suitable CTAB/Fe^3+^ ratio.

## Conclusions

In this study, based on the structural, textural, and elemental characteristics' a possible magnetic microstructure of barium hexaferrite particles was discussed. Barium hexaferrite properties highly depended on the synthesis conditions, especially during precipitation with the addition of CTAB surfactant. For a fixed amount of surfactant and Fe^3+^/Ba^2+^ ratio, an increase in ions concentration resulted in the formation of bigger grains and higher content of the barium-rich phase in a final product. Obtained series of pure BaM samples exhibited significant changes in elemental composition, allowing for the formation of both iron and barium excessive single-phase materials. Meanwhile, the surplus iron can be incorporated into the BaFe_12_O_19_ structure, while barium tends to accumulate on the grain surface despite the overall Fe/Ba ratio. It suggests that the grain boundary of as-synthesized ferrite could possess properties different than bulk BaM. For the samples obtained at a barium rich environment and with a high CTAB/Fe^3+^ ratio, a significant softening of the final material was observed, connected to both decreased grain size and the possible effect of dipolar interactions, together with a formation of Ba-rich layer at the surface. The overall approach allowed us to synthesize a series of BaFe_12_O_19_ materials with behaviour similar to superparamagnetic transition, despite being far from the theoretical point of superparamagnetic critical size. This study provides a new method for tailoring magnetic properties of barium hexaferrite, where high magnetization is preserved, and additional elements are not required to modify the crystal structure of the material.

## Conflicts of interest

There are no conflicts to declare.

## Supplementary Material

RA-010-D0RA01619E-s001
