# Supplementary material for: Pseudo-superparamagnetic behaviour of barium hexaferrite particles
Source: RSC Adv. 2020 May 18;10(32):18784–96. doi: 10.1039/d0ra01619e (PMC9053871; doi:10.1039/d0ra01619e)
Supplement: RA-010-D0RA01619E-s001 [file RA-010-D0RA01619E-s001.pdf]

Electronic Supplementary Material (ESI) for RSC Advances.
This journal is © The Royal Society of Chemistry 2020

Supporting Information:
Pseudo-superparamagnetic behaviour of barium hexaferrite particles

Szymon Dudziak,*[a] Zuzanna Ryżyńska,[b] Zuzanna Bielan,[a] Jacek Ryl,[c] Tomasz Klimczuk,[b] Anna Zielińska-Jurek [a]

[a] *Department of Process Engineering and Chemical Technology, Gdansk University of Technology, G. Narutowicza 11/12, 80-233 Gdansk, Poland.*
[b] *Department of Solid State Physics, Gdansk University of Technology, G. Narutowicza 11/12, 80-233 Gdansk, Poland.*
[c] *Department of Electrochemistry, Corrosion and Materials Engineering, Gdansk University of Technology G. Narutowicza 11/12, 80-233 Gdansk, Poland.*
* Correspondence: dudziakszy@gmail.com.

Table S1. shows obtained a and c lattice constants together with the calculated unit cell volume of $BaFe_{12}O_{19}$ samples. Based on the obtained values, the densities of synthesized materials were calculated. For samples BaM_10_1, BaM_12_2 and BaM_12_3 obtained values were used to recalculate magnetization from mass one [$Am^2 \cdot kg^{-1}$] to volume [$A \cdot m^{-1}$] during law of approach magnetization to saturation (LAMS) calculations.

Table S1. Unit cell information for the obtained samples and calculated densities.

Sample	BaM lattice const. *a* [Å]	BaM lattice const. *c* [Å]	BaM unit cell volume [Å^3]	Density [$kg \cdot m^{-3}$]
BaM_8_1	5.894	23.279	700.245	5271.31
BaM_8_2	5.894	23.214	698.387	5285.33
BaM_8_3	5.888	23.190	696.333	5300.92
BaM_8_3D	5.886	23.186	695.726	5300.06
BaM_10_1	5.887	23.203	696.446	5282.26
BaM_10_1D	5.897	23.231	699.563	5321.42
BaM_10_1C	5.897	23.258	700.388	5365.51
BaM_10_2	5.892	23.075	693.651	5344.72
BaM_10_3	5.867	23.075	687.950	5272.64
BaM_10_4	5.873	23.122	690.627	5291.55
BaM_12_1	5.894	23.273	700.069	5281.14
BaM_12_2	5.892	23.198	697.566	5276.55
BaM_12_3	5.894	23.230	698.942	5305.55
BaM_12_4	5.897	23.231	699.550	5276.44
BaM_12_4C	5.892	23.219	698.038	5271.31

XRD results of samples BaM_12_3 and BaM_12_4 after original 2h calcination are presented in Figure S1.

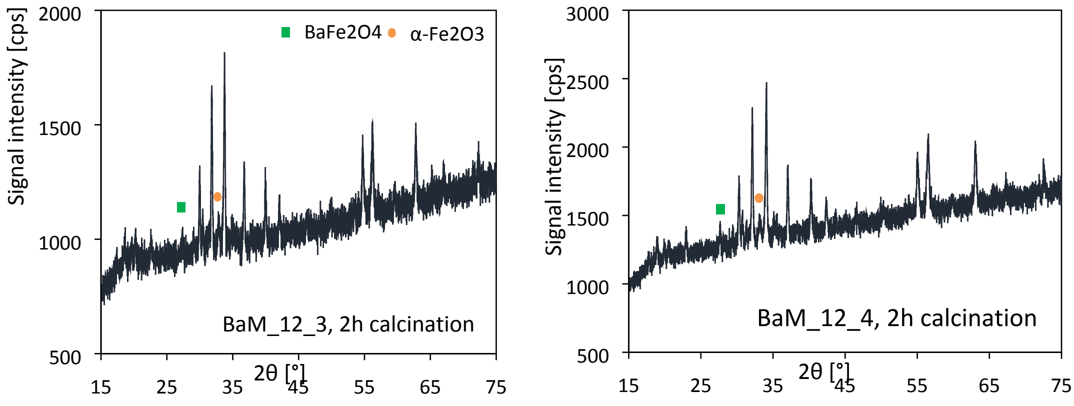

Figure S1. XRD patterns of samples BaM_12_3 and BaM_12_4 before additional calcination

Additional SEM images of samples BaM_10_1, BaM_12_2 and BaM_12_3 are shown in Figure S2.

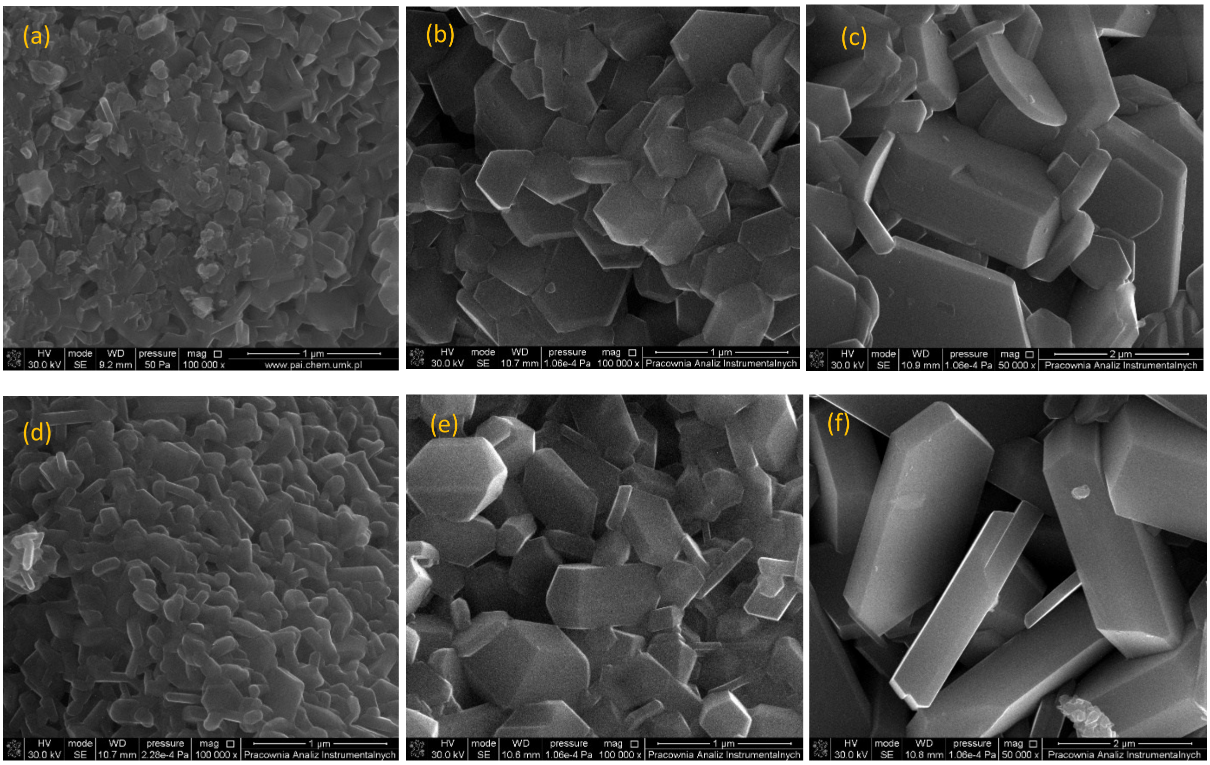

Figure S2. SEM images of the selected $BaFe_{12}O_{19}$ samples: BaM_10_1 (a,d), BaM_12_2 (b,e), and BaM_12_3 (c,f)

Observed particles for sample BaM_10_4, produced from concentrated solution with a Fe^{3+}/Ba^{2+} = 10, similar as sample BaM_10_1 is shown below in Figure S3, proving critical role of the surfactant on the grains size and their distribution.

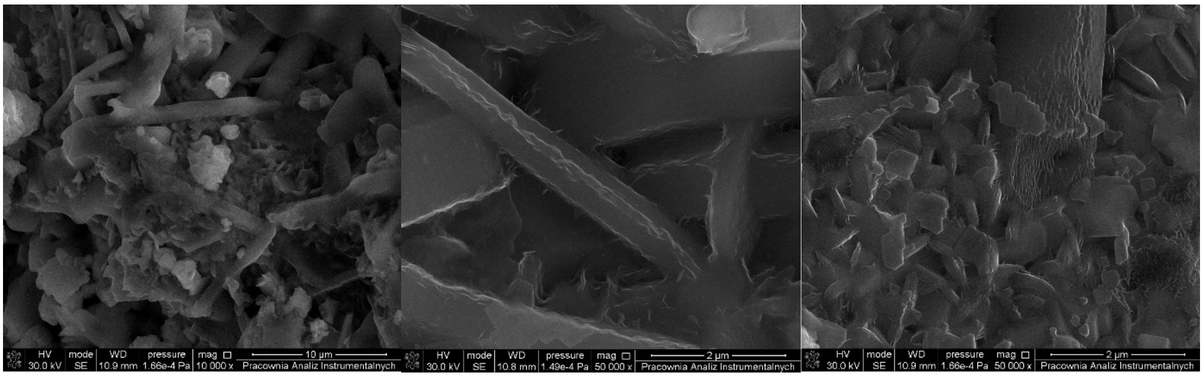

Figure S3. SEM images of BaM_10_4 sample

The formation of the BaM phase during calcination is presented in Figure S4. Obtained results showed that $BaFe_{12}O_{19}$ formation starts to occur between 600°C and 700°C from the initially amorphous precursor. The exact identification of the intermediate phase visible at 600°C was not performed. For the sample calcined at 700°C, all characteristic signals corresponding to the BaM phase are observed.

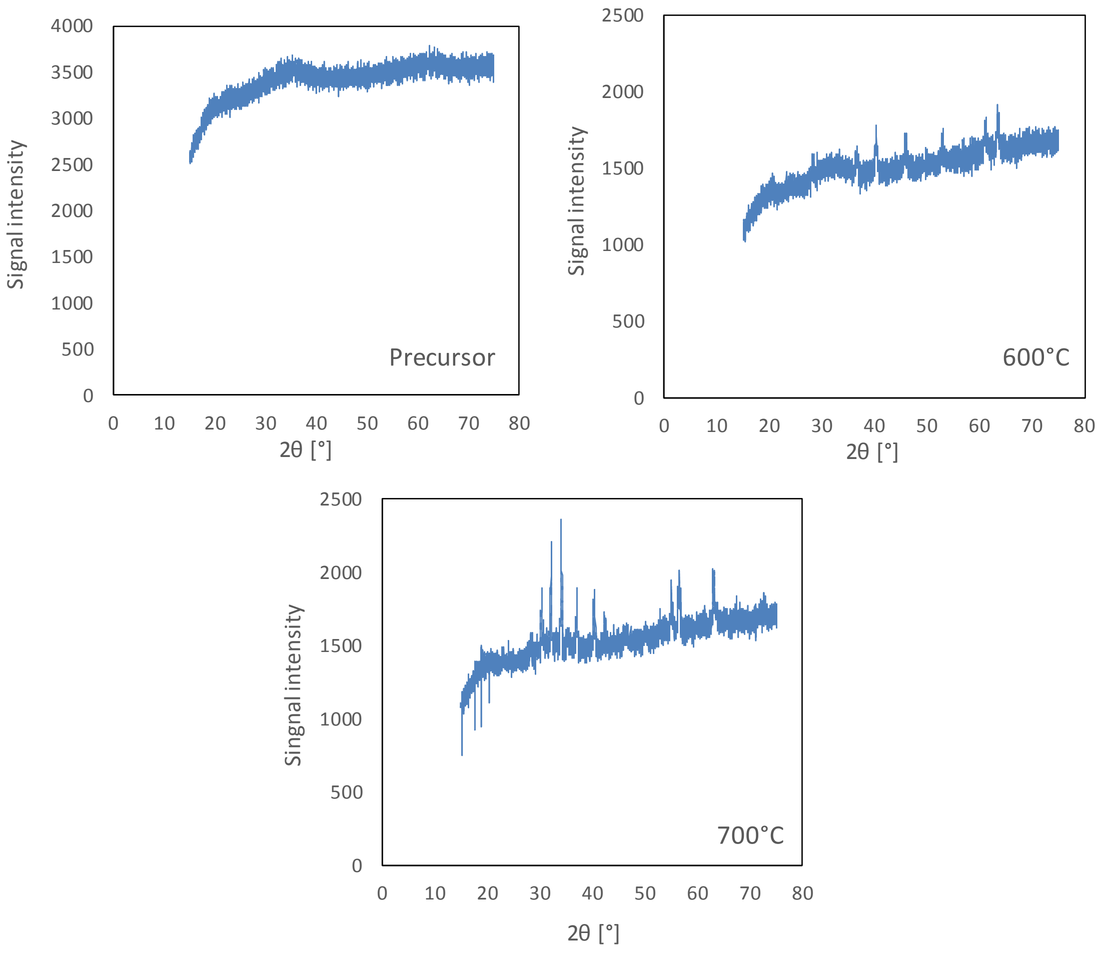

Figure S4. XRD patterns of an exemplary precursor and its phase evolution during the calcination process

All the obtained magnetic hysteresis loops are shown in Figure S5, together with their zoom-ins at the centre for the selected samples. The specific values of saturation, remanence, and coercivity are presented in Table 1 in the main text.

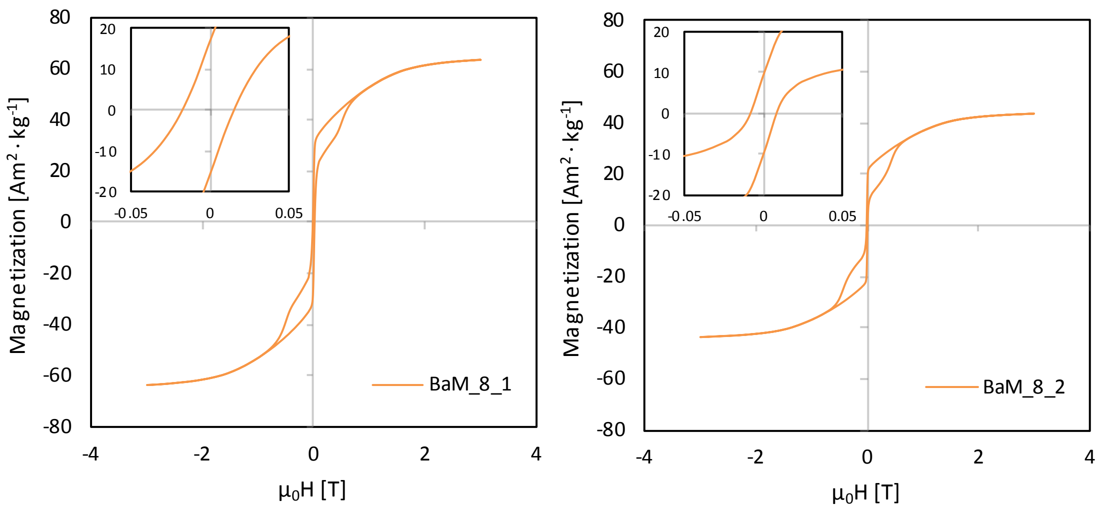

Figure S5. The obtained hysteresis loops for the barium hexaferrite samples

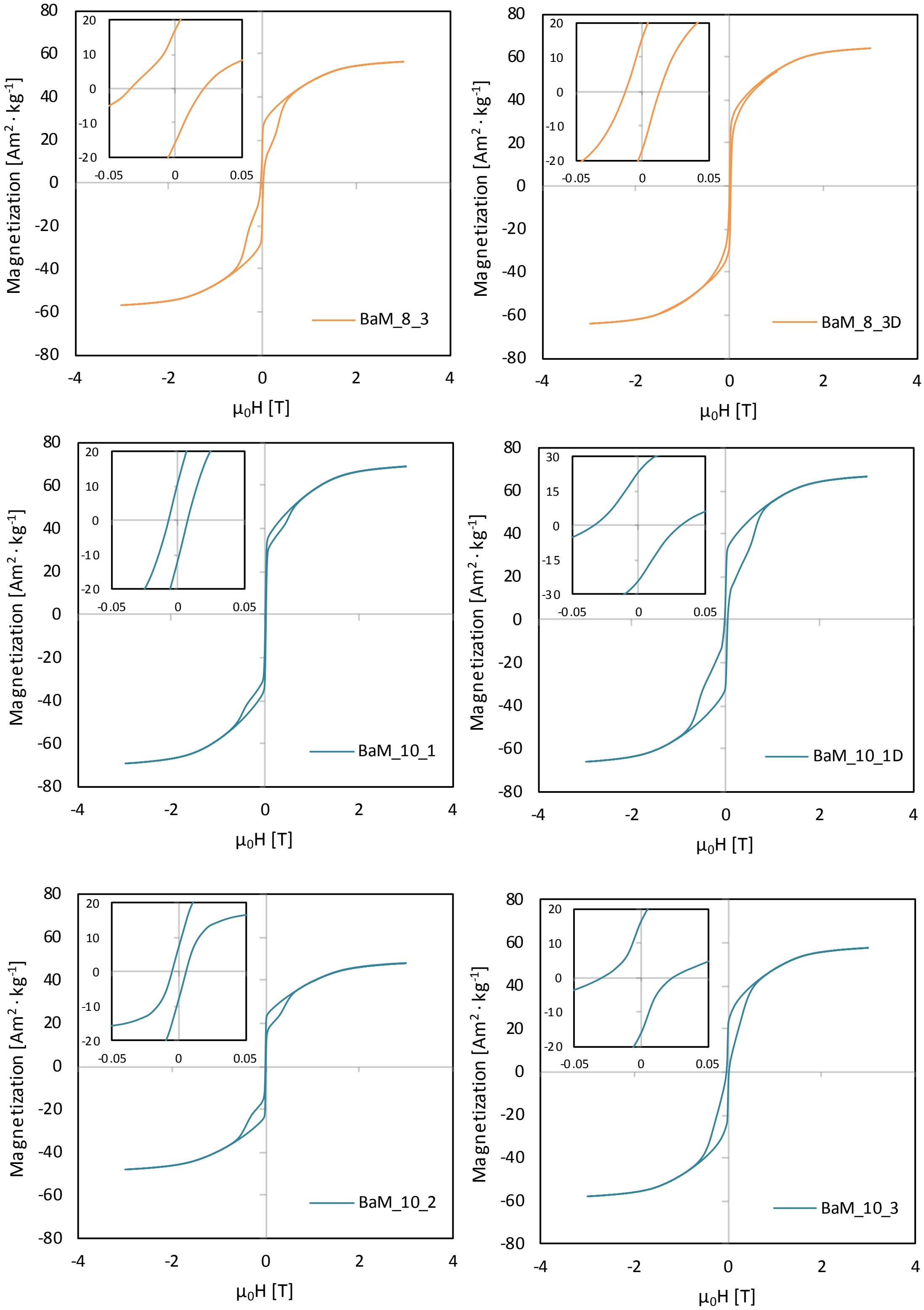

Figure S5. cont. The obtained hysteresis loops for the barium hexaferrite samples

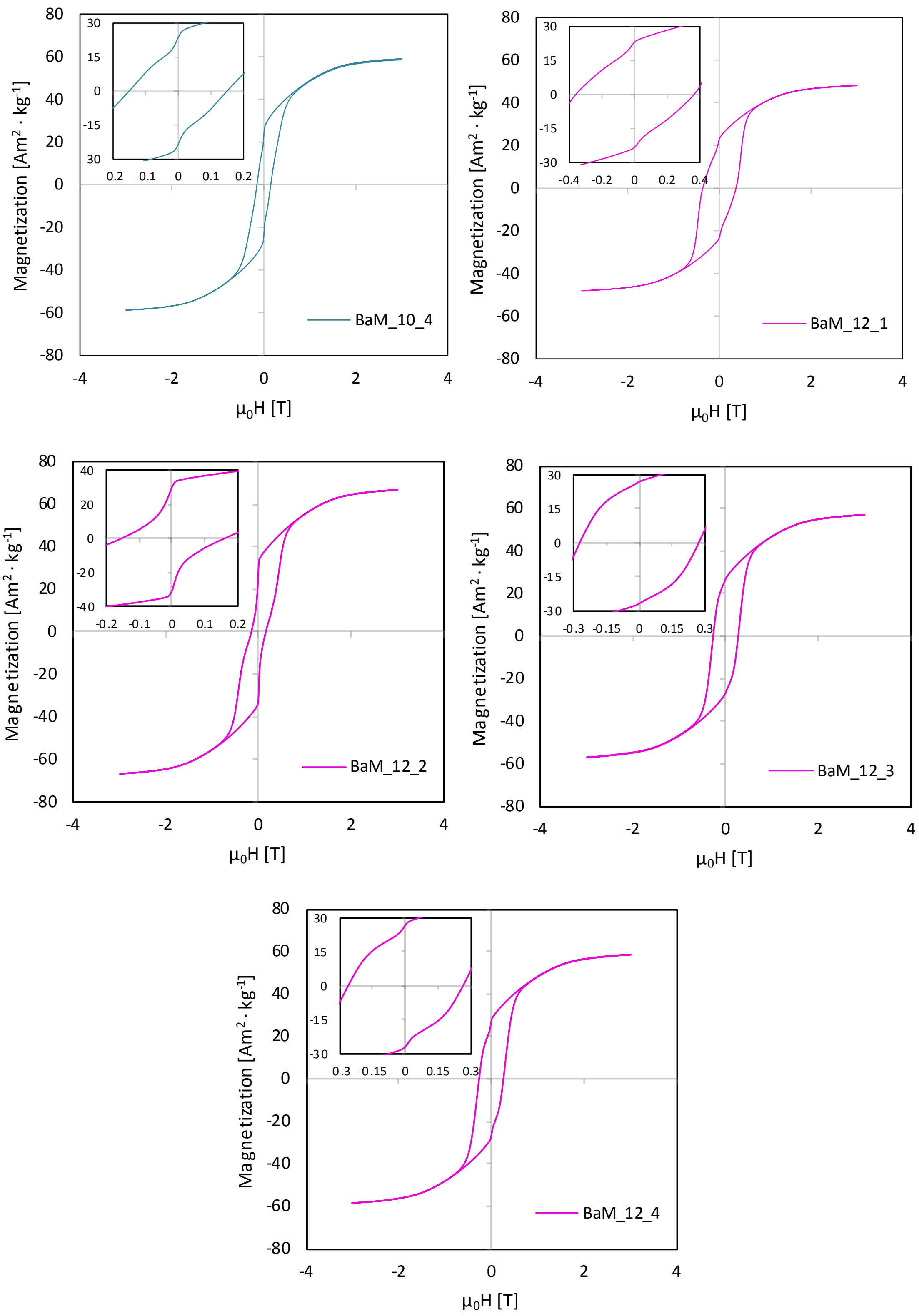

Figure S5. cont. The obtained hysteresis loops for the barium hexaferrite samples

Effective anisotropy constants for selected samples were obtained using LAMS calculations. In this regard, magnetization data was converted from mass magnetization to volume magnetization based on materials density obtained from XRD results. Obtained volume magnetization data were then fitted to a known equation:

$$M(H) = M_S \cdot \left(1 - \frac{a}{H} - \frac{b}{H^2}\right) + \chi H$$

where M is magnetization, M_S is magnetic saturation, H is magnetic field strength, χ is high field magnetic susceptibility and a, b are numerical parameters to be found. M_S value was adapted from PPMS measurements and analysis was performed in the first quadrant of the hysteresis in the region of $M \geq 0.9 \cdot M_S$. The results of performed fitting are shown in Figure S6. The mean absolute percentage error (MAPE) of the fitting was calculated using formula:

$$MAPE = \frac{\sum_{i=1}^{n} \frac{\left|Experimental_i - Model_i\right|}{Experimental_i}}{n} \cdot 100\%$$

where *Experimental* and *Model* are magnetization values obtained from measurements and fitting respectively and *n* is number of fitted points. For performed fitting MAPE values were lower than 0.1% and are shown in Figure S4.

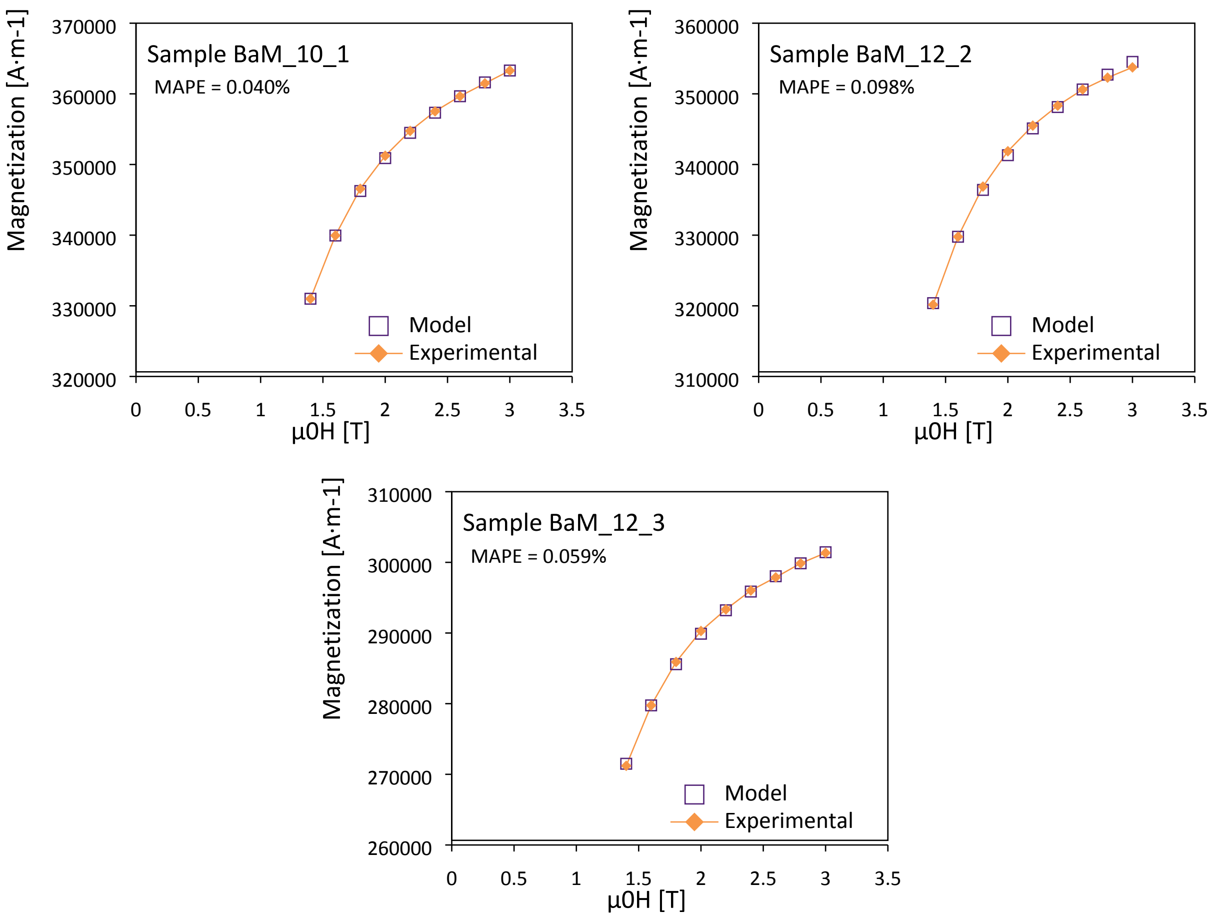

Figure S6. Graphical results of LAMS fitting to experimental data

The differential dM/dH curve for sample BaM_10_4 is shown in Figure S7, with a sample BaM_10_1 for comparison. It was found that BaM_10_4 sample possessed visibly less coupled character than BaM_10_1, which is also connected with a visible increase in M_R value.

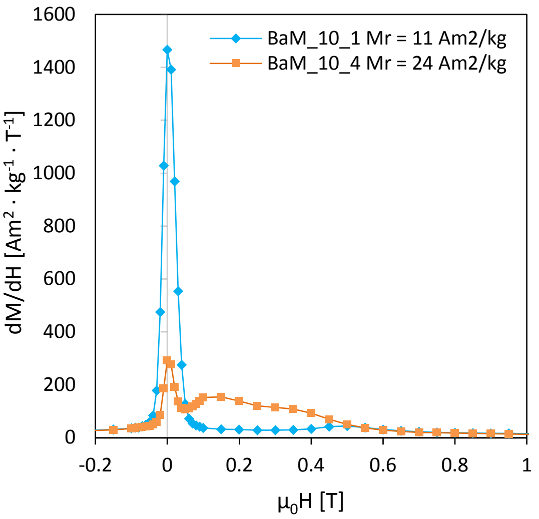

Figure S7. Comparison of the dM/dH curves for the samples BaM_10_1 and BaM_10_4

Basic information about re-synthesized sample BaM_10_1 is shown below. The re-synthesized sample was found to be pure $BaFe_{12}O_{19}$ with a little less crystalline structure than the original one, as shown in Figure S8.

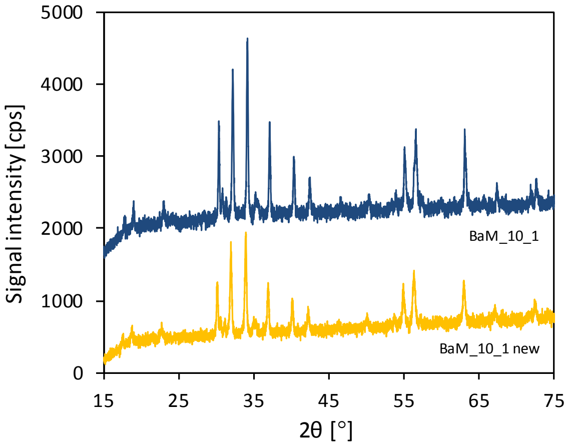

Figure S8. XRD patterns of re-synthesized BaM_10_1 sample (yellow) and the original one (blue)

Morphology and distribution of the observed grains' height and width are shown in Figures S9 and S10, respectively.

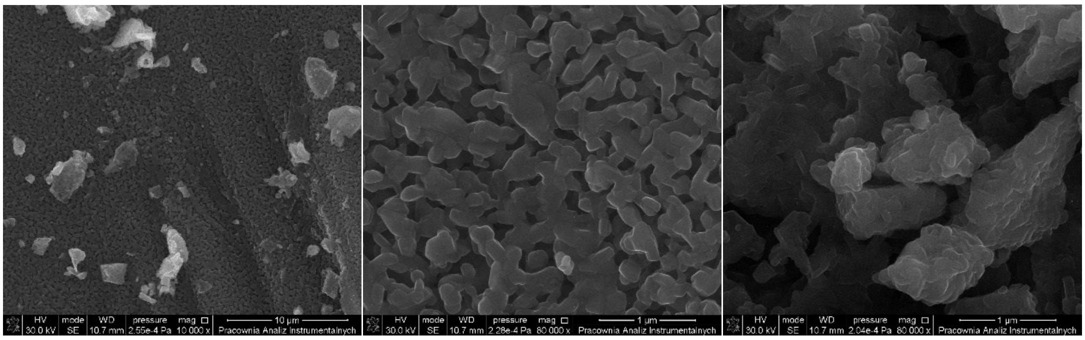

Figure S9. The obtained SEM images of re-synthesized sample BaM_10_1.

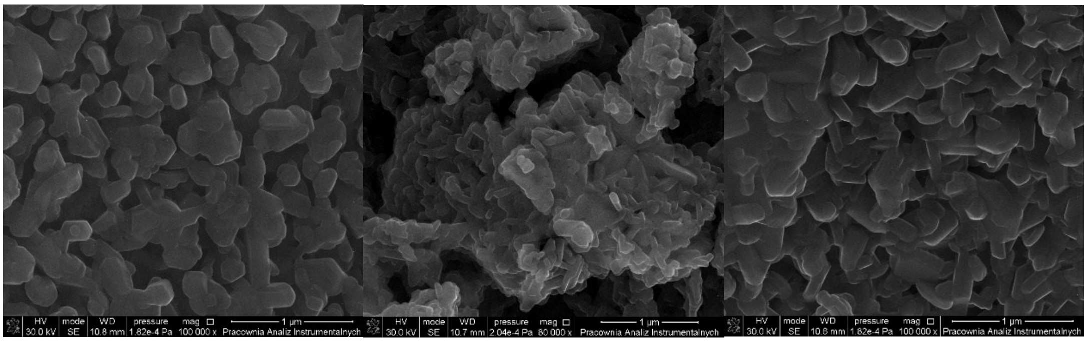

Figure S9 cont. The obtained SEM images of re-synthesized sample BaM_10_1.

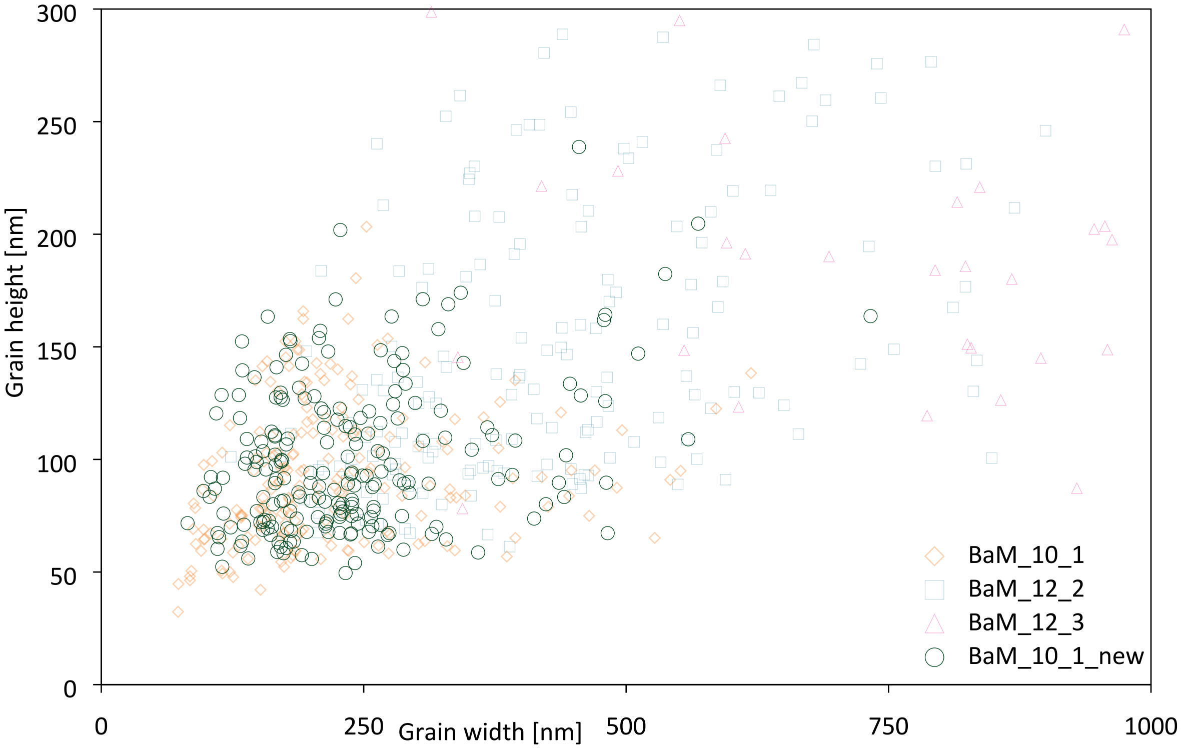

Figure S10. Size distribution of observed grains for re-synthesized sample BaM_10_1 and previous results from Figure 5 in the main text.

Magnetic properties of the new BaM_10_1 sample are shown in Figure S11a, together with LAMS fitting results shown in Figure S11b. It is seen that the main differences are observed in H_C value and a parameter during LAMS analysis. The summation of the magnetic properties of BaM_10_1_new is shown in Table S2.

Table S2. Results of magnetic properties analysis for sample BaM_10_1_new.

Sample	M_S [$Am^2 \cdot kg^{-1}$]	M_R [$Am^2 \cdot kg^{-1}$]	M_R/M_S	H_C [$kA \cdot m^{-1}$]	a [T]	b [T^2]	K [$J \cdot m^{-3}$]	χ [$A \cdot T^{-1} m^{-1}$]
BaM_10_1	69	11	0.16	6.0	0	0.1940	$3.10 \cdot 10^5$	2613
BaM_10_1_new	69	26	0.38	71.0	0.0107162	0.19255	$3.08 \cdot 10^5$	2999.903

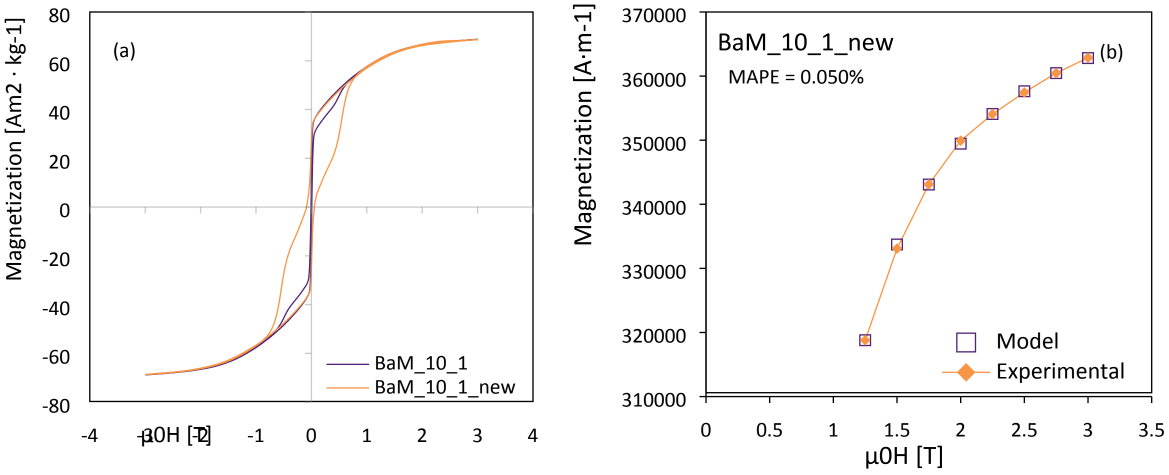

Figure S11. Magnetic hysteresis loops for the obtained BaM_10_1_new sample (a) and results of performed LAMS fitting (b).
